# Combination of M2e peptide with stalk HA epitopes of influenza A virus enhances protective properties of recombinant vaccine

**DOI:** 10.1371/journal.pone.0201429

**Published:** 2018-08-23

**Authors:** Liudmila M. Tsybalova, Liudmila A. Stepanova, Marina A. Shuklina, Eugenia S. Mardanova, Roman Y. Kotlyarov, Marina V. Potapchuk, Sergei A. Petrov, Elena A. Blokhina, Nikolai V. Ravin

**Affiliations:** 1 Department of Vaccinology, Smorodintsev Research Institute of Influenza, Ministry of Health of the Russian Federation, St. Petersburg, Russia; 2 Institute of Bioengineering, Research Center of Biotechnology, Russian Academy of Sciences, Moscow, Russia; Deutsches Primatenzentrum GmbH - Leibniz-Institut fur Primatenforschung, GERMANY

## Abstract

**Background:**

Influenza infection could be more effectively controlled if a multi-purpose vaccine with the ability to induce responses against most, or all, influenza A subtypes could be generated. Conserved viral proteins are a promising basis for the creation of a broadly protective vaccine. In the present study, the immunogenicity and protective properties of three recombinant proteins (vaccine candidates), comprising conserved viral proteins fused with bacterial flagellin, were compared.

**Methods:**

Balb/c mice were immunized intranasally with recombinant proteins comprising either one viral protein (the ectodomain of the M2 protein, ‘M2e’) or two viral proteins (M2e and the hemagglutinin second subunit ‘HA2’ epitope) genetically fused with flagellin. Further, two different consensus variants of HA2 were used. Therefore, three experimental positives were used in addition to the negative control (Flg-his). The mucosal, humoral, and T-cell immune responses to these constructs were evaluated.

**Result:**

We have demonstrated that insertion of the HA2 consensus polypeptide (aa 76–130), derived from either the first (HA2-1) or second (HA2-2) virus phylogenetic group, into the recombinant Flg4M2e protein significantly enhanced its immunogenicity and protective properties. Intranasal administration of the vaccine candidates (Flg-HA2-2-4M2e or Flg-HA2-1-4M2e) induced considerable mucosal and systemic responses directed at both the M2e-protein and, in general, the influenza A virus. However, the immune response elicited by the Flg-HA2-1-4M2e protein was weaker than the one generated by Flg-HA2-2-4M2e. These recombinant proteins containing both viral peptides provide complete protection from lethal challenge with various influenza viruses: A/H3N2; A/H2N2; and A/H5N1.

**Conclusion:**

This study demonstrates that the intranasal administration of Flg-HA2-2-4M2e recombinant protein induces a strong immune response which provides broad protection against various influenza viruses. This construct is therefore a strong candidate for development as a universal vaccine.

## Introduction

Influenza A epidemic and pandemic control is one of the principle problems of contemporary medicine. It can potentially be solved through the creation of a broadly protective influenza vaccine. The range of such (universal) vaccines extends from vaccines directed at different strains within one virus subtype to vaccines against both A and B influenza virus types. In recent years, significant progress has been made in the development of universal vaccines [1–10] and some of them have undergone phase II, III clinical trials [1, 2]. Nevertheless, the search of an optimal vaccine composition continues [7, 8].

Broad protection against severe form of influenza A is provided by vaccines based on conserved viral proteins. Conserved surface-exposed antigens, such as the extracellular domain of the M2 protein (M2e) and the stalk region of hemagglutinin (HA2), are the most promising targets for vaccine formulations that can protect against multiple influenza A virus subtypes [4, 6, 8, 10].

The M2 protein forms an ion channel in the viral envelope and plays important roles in acid-induced proton gating and in further interactions between the viral nucleoprotein complex (vRNPs) and M1 after penetration [11]. The M2 ion channel is a tetramer of individual subunits, and viral particles have from 1 to 3 surface channels. The ectodomain of the M2 protein (23 aa) is not available to effectors of the immune system, as it is hidden by the larger surface proteins HA (≈ 567 aa) and NA (≈ 464 aa). Despite this, it has two promising features. First, M2e is completely conserved at its 9 N-terminal amino acids; they are identical among all influenza A virus subtypes (H1-H18). It has minor mutations in the distal portion [12]. Second, this protein is abundantly present on the surfaces of infected cells, and it is currently considered a promising target for immune system effectors. Due to its presence on the surfaces of infected cells, it readily binds to specific immunoglobulin receptors, thereby causing Fc-receptor-mediated antibody-dependent cytotoxicity (ADCC) and antibody-dependent phagocytosis (ADPC) [10]. Another mechanism is complement-dependent cytolysis (CDC). It has been shown that although M2e-based vaccine candidates do not prevent diseases, they do reduce clinical symptoms and prevent deaths in experimental animals [5, 12, 13].

It is generally assumed that introducing additional conserved viral epitopes, capable of inducing neutralizing antibodies, into recombinant proteins used for immunization should lead to enhanced protective effects [8]. The second subunit of hemagglutinin (HA2) is one such possible candidate. It is relatively conserved within viral strains from the same phylogenetic group (I or II). HA2 is responsible for the fusion of viral and cellular membranes in endosomes, thereby allowing ribonucleic complexes into the cytoplasm [14]. Neither immunization with traditional vaccines, nor natural influenza infection lead to the formation of a significant amount of anti-HA2 antibodies; this is due to its low immunogenicity in the presence of immunodominant regions of receptor-binding HA1 [15]. However, in the last decade, a number of monoclonal antibodies (from mice and humans) capable of reacting with HA2 epitopes have been highlighted [16–25]. These antibodies are cross-reactive, and they bind or neutralize influenza virus subtypes within one phylogenetic group, thus providing a wide range of protection. Furthermore, some antibodies, such as F16 and MED18852, bind epitopes from both groups I and II [22, 24, 25]. Anti-HA2 antibodies are less potent at direct viral neutralization as compared to anti-head (HA1), but it has been shown that they can induce potent ADCC of infected cells [26]. In recent years, a number of candidate vaccines based on the hemagglutinin stalk epitopes of influenza A viruses belonging to phylogenetic groups I and II (amino acids: 38–59; 23–185; 76–130) have been developed [6, 27–31].

In the HA2 native molecule, the amino acid region 76–130 forms an alpha-helix at the 75–125 position. After attachment to the receptor on the target cell, virus is internalized by receptor-mediated endocytosis. At low pH in the endosome this part of HA2 (aa76-130) is restructured and its conformation is changed. New structure consist of a part of long alpha-helix (aa 38–106); a loop (aa 106–112); and a short helix (aa 112–125). The conformational changes are reflected in an increased reactivity of monoclonal antibodies (mAbs) recognizing corresponding HA2 epitopes [29]. The 12D1 mouse mAb binds amino acids within the highly conserved long alpha-helix region of HA2 from virus subtype H3 -more precisely, at the 76–106 site [27]. Later, the authors found that the best binding is provided by a structure comprising amino acids 76–130. A candidate vaccine, based on this peptide coupled to the carrier protein keyhole limpet hemocyanin (KLH), was shown to have activity against influenza viruses from both phylogenic groups I and II (H3, H5, H1) [27]. Both M2e-elicited and HA-stalk-elicited serum antibodies have been shown to confer protection through passive transfer [17, 27, 32, 33].

However, both the M2e and the HA polypeptide (aa 76–130) are weakly immunogenic in their native forms without modification, thus investigators have used different ways to improve their immunogenic properties [4, 5, 12, 32]. One approach is the genetic construction of hybrid proteins that fuse partial peptides to flagellin. Bacterial flagellin, as a natural ligand of Toll-like receptor 5 (TLR5), is a prospective adjuvant and a carrier for vaccine proteins [34–38]. It is able to stimulate an effective immune response to influenza A viral proteins such as some domains of HA1 [38] or the weakly immunogenic M2e peptide [34, 37]. It activates TLR5-positive DCs, neutrophils, or epithelial cells of the respiratory compartment [39, 40], and it stimulates the signaling of TLR5 in lung epithelial cells and pneumonocytes [41]. Flagellin causes a massive recruitment of granulocytes and macrophages/monocytes in the airways and the production of inflammatory mediators [42]. This robust inflammation is necessary for the initiation of strong cellular and humoral responses. One of the advantages of flagellin is its efficiency as an adjuvant for intranasal administration.

In our previous studies, it was shown that intranasal immunization of mice with recombinant flagellin containing 4 copies of the M2e peptide at the C-terminus provides an effective immune response and protects mice from lethal influenza infection [37]. In this study, we enhanced the protective effect of the vaccine candidate by including in the flagellin fusion protein both the M2e peptide and the HA stalk domain (aa 76–130).

## Materials and methods

### Sequence analysis of HA2 from different influenza A viruses

Amino acid sequences (aa 76–130, H3 numbering) of influenza A viral HA2 were taken from the GenBank and GISAID databases. In order to construct consensuses, the amino acid sequences were aligned using the MAFFT server with the FFT-NS-i, or FFT-NS-2 algorithms (depending on the number of sequences) [43], and analyzed in Unipro UGENE v.1.14.0. Alignment and analysis of a small number of sequences was carried out in the Vector NTI software package (Invitrogen, USA). The search for experimental B and CD4+ T-cell epitopes homologous to sections of HA2 was performed in the Immune Epitope Database [44]. The search for CD8+ T-cell epitopes was performed using the NetCTLpan1.1 Server [45] with default search parameters. The image of the three-dimensional structure of HA was obtained in the UCSF Chimera v.1.9 program [46] using the 4JTV (4O5I (A/Victoria/06/2011 (H3N2)) models taken from the RCSB Protein Data Bank database. Visualization of the three-dimensional protein structure was performed in the Chimera 1.5.3 program [46]. The open web resource Phyre2 was used for homologous modeling of protein three-dimensional structure [47].

### Design and expression of recombinant proteins

**Fig 1:**
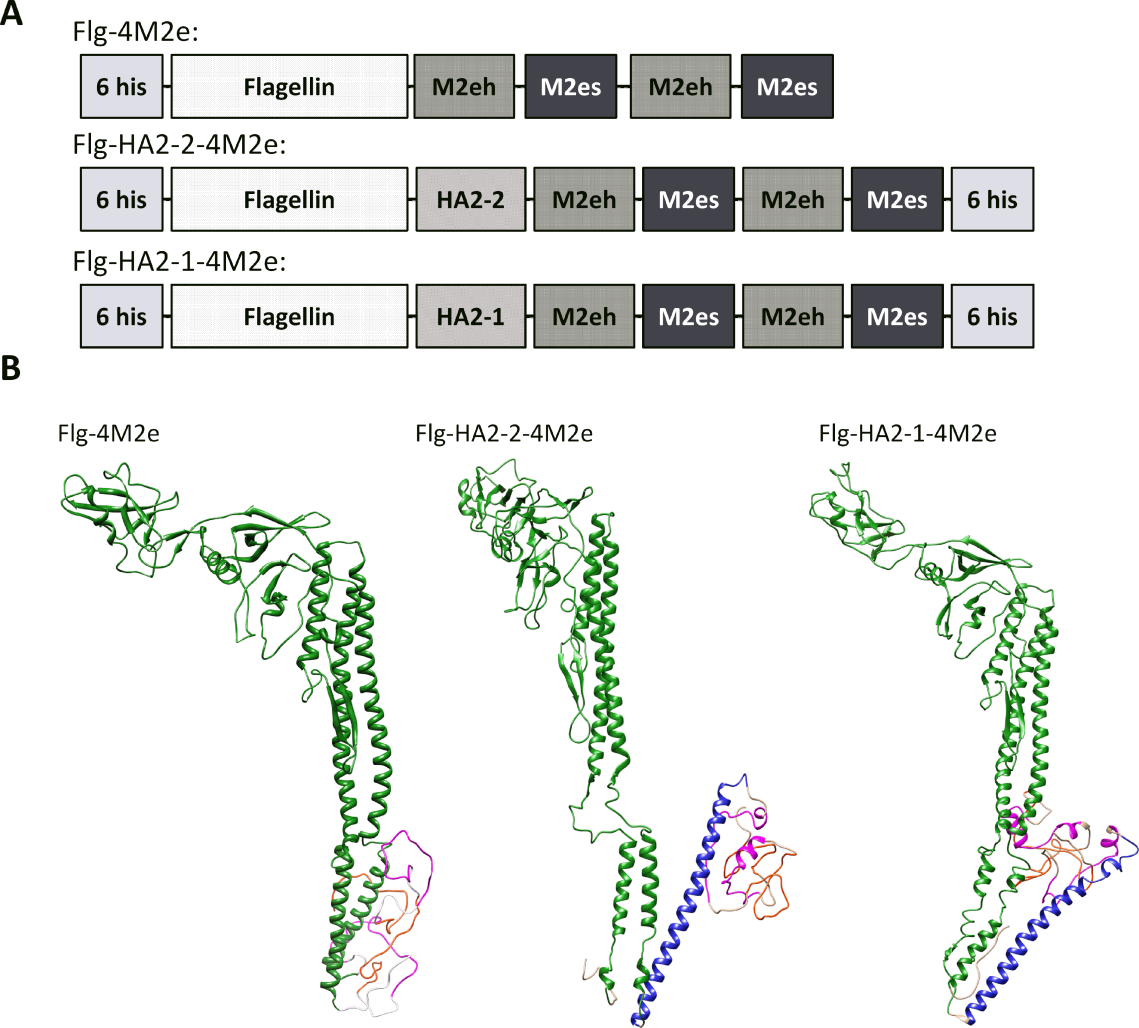
Construction of recombinant proteins. **(A) Schematic representation and (B) theoretical model of the 3D-structure of the monomeric recombinant fusion proteins.** Boxes are not drawn to scale. Yellow marks M2es; pink marks M2eh; blue marks HA2 fragment; and green is flagellin. Modeling was performed with Phyre 2 server, and visualization with USCF Chimera.

Four chimeric genes were constructed using genetic engineering techniques. One of them encoded full-length flagellin (Flg) amplified from the genomic DNA of *Salmonella typhimurium* (nucleotide sequence ATU59429.1, GenBank). A second gene encoded full-length flagellin with sequence encoding four tandem copies of the M2e peptide at the C-terminus added. The four M2e copies were comprised of two types. Two copies (M2eh) were the consensus peptide sequence representing M2e from human virus subtypes A/H1N1, A/H2N2, and A/H3N2. The other two copies (M2es) were sequence from influenza virus A/H1N1pdm09. These copies were arranged as M2eh- M2es- M2eh- M2es. Two point mutations, C16S and C18S, were made in the M2e sequence (Table 1). The M2e sequences were separated from each other by glycine-rich linkers (GGGSG). The third gene encoded the same Flg and M2e proteins, but with an additional fragment, HA2-2, which is a consensus (aa 76–130) of the HA stalk from the A/H3N2 and A/H7N9 influenza viruses (phylogenetic group II-d) (S1 Fig). In the fourth gene, the HA2-2 sequence was replaced with HA2 representing a consensus of several phylogenetic group I-t viruses (A/H1N1, A/H1N1pdm, A/H2N2, A/H5N1); we term this portion ‘HA2-1’ (S2 Fig). These are detailed in Table 1. To summarize, three recombinant proteins were designed (**Fig 1**).

**Table 1:** Influenza A virus derived amino acid sequences used.

**M2 protein ectodomain (aa 2–24)**	
M2eh (consensus)	SLLTEVETPIRNEWGCRCNDSSD
M2es (A/H1N1pdm09)	SLLTEVETPTRSEWECRCSDSSD
**HA second subunit (aa 76–130)**	
HA2-2 (consensus)	RIQDLEKYVEDTKIDLWSYNAELLVALENQHTIDLTDSEMNKLFEKTRRQLRENA
HA2-1 (consensus)	RLENLNKKMEDGFLDVWTYNAELLVLMENERTLDFHDSNVKNLYDKVRMQLRDNA

Each of the synthesized genes was cloned into the pQE30 (Qiagen) expression vector at its BamHI and KpnI sites. All of the recombinant proteins contained a histidine tag at their N-termini. A 6-histidine tag was added to the C-termini of the constructs in order to provide better binding ability (to the Ni-NTA sorbent). Thus, the vectors (pQE30/Flag, pQE30/Flag4M2e, pQE30/Flag4M2eHA2-2, and pQE30/Flag4M2eHA2-1) were assembled, and *Escherichia coli* (*E*. *coli*) DLTI270 cells were transformed with one of the plasmids for the expression of the corresponding recombinant protein. The DLT1270 strain, a derivative of DH10B, contains a *lacI* lactose operon repressor gene integrated into the chromosome. The strains were grown at 37°C in LB medium, supplemented with 100 μg/ml of ampicillin, until the midpoint of the logarithmic growth phase (OD600 = 0.4–0.7). Next, IPTG was added, to a 0.1 mM endpoint concentration, and the culture was grown for a further 3–12 h at 30°C.

### Purification of recombinant proteins

Following induction, the bacteria were collected by centrifugation and resuspended. This suspension was supplemented with 1mM PMSF and treated with 1 mg/ml lysozyme for 15 min on ice, followed by sonication. The lysate was then centrifuged at 13,000 g for 5 min. Recombinant proteins were purified from the cell lysate using metal affinity chromatography; they were bound on a Ni-sorbent (GE, USA), equilibrated with 20 mM phosphate buffer, pH 8.0, containing 5 mM imidazole, for 60 min. Following binding of the target proteins, resins were washed with 20 mM phosphate buffer, pH 8.0, containing 20 mM imidazole. The recombinant proteins were eluted with 20 mM phosphate buffer, pH 8.0, containing 0.5 M imidazole and subsequently dialyzed against 10 mM phosphate buffer, pH 7.2 (PBS).

### SDS-PAGE and Western blotting

The expressed recombinant proteins were separated on 8–16% gradient polyacrylamide gels under denaturing conditions, followed by staining with Coomassie brilliant blue (R-250) for 18 hours. For Western blotting, after completion of electrophoresis, the proteins were then transferred to a nitrocellulose membrane in TB (blot transfer buffer) using the Mini Trans blot system (Bio-Rad, USA). The membrane was blocked with 3% BSA at room temperature overnight and then incubated with 14C2 mouse anti-M2e monoclonal antibody (ab5416, Abcam, UK) or rabbit polyclonal antibodies specific for flagellin (ab93713, Abcam, UK). Binding signals were visualized by staining the membrane with secondary antibodies for 1 h at room temperature. Either peroxidase-labelled goat anti-rabbit IgG (Invitrogen, USA) or goat anti-mouse IgG (Invitrogen, USA) were used. Development was via incubation in TMB immunoblot solution (Invitrogen, USA) for 5 min.

### Ethics statement

The study was carried out in strict accordance with the Russian Guidelines for the Care and Use of Laboratory Animals (1977). The protocol was approved by the Committee for Ethics of Animal Experimentation of the Research Institute of Influenza (Permit Number: 1701). All efforts were made to minimize animal suffering. Mice were housed in cages provisioned with water and standard food and monitored daily for health and condition. A loss of body weight exceeding 30% was used as a criterion for early euthanasia. The animals were euthanized by CO_2_ inhalation for 5 minutes.

### Mice and immunization

Specific pathogen free, female BALB/c mice (16–18 g) were purchased from the "Stolbovaya" (Moscow region) animal facility. Mice were immunized intranasally (i.n.) on days 0 (primary vaccination), 14 (first boost), and 28 (second boost) with 10 μg/0.02ml (5 μg each nostril) of one of the proteins under inhalation anesthesia conditions (2–3% isoflurane mixed with 30% oxygen (O_2_) and 70% nitrous oxide (N_2_O)). The three peptides were: Flg-4M2e (M2eh-M2es-M2eh-M2es); Flg-HA2-2-4M2e; or Flg-HA2-1-4M2e. Control mice were sham immunized i.n. with 0.02 ml of PBS or Flg-his.

### Sample collection

Blood, bronchoalveoloar lavages (BAL), and nasal wash samples were collected from 5 mice in each group on day 14 after the second-boost immunization. Mice from the experimental and control groups (5 mice from each group) were euthanized, using a CO_2_ chamber, on the 14th day after the second boost. Blood samples were incubated at room temperature for 30 min, and the sera were obtained by centrifugation for 15 min at 4,000 g. To expose the trachea, mice were dissected, and an IV catheter (BD Bioscience) was then inserted into a small nick in the trachea. BAL samples were collected via a 2-fold flushing of the airway compartment with 500 μl of PBS. The samples were stored at −20°C until use.

Two weeks after immunization, mouse lungs were removed aseptically and placed into eppendorf tubes with RPMI-1640 containing 0.5 mg/ml collagenase (Sigma, C2674) and 25 μg/ml DNAse (Sigma, D4263). Lungs were homogenized using a Tissue Lyser II, placed into a thermo-shaker (45 min, 37°C), and purified of cell debris by filtration through a syringe filter with a 70 μm pore size (Syringe Filcons, BD Biosciences, USA). Erythrocytes were lysed with ACK lysing buffer (0.15 M NH_4_Cl, 1.0 M KHCO_3_, 0.1 mM Na_2_EDTA, pH 7.2–7.4). Lung cells were washed with complete RPMI-1640 medium containing 10% FBS, 2 mM L-glutamine, 100 U/mL of penicillin, and 100 μg/ml of streptomycin. The cell concentration was adjusted to 5×10^6^ cells/ml.

### Synthetic peptides

The following peptides (synthesized by Verta, Russia) were experimentally tested: M2es SLLTEVETP**T**R**S**EW**E**CRC**S**DSSD (M2e of A/California/07/09 H1N1pdm09); and M2eh SLLTEVETP**I**R**N**EW**G**CRC**N**DSSD (consensus M2e of human influenza A viruses). Residues that differ between the sequences are displayed in bold font and underlined.

### Viruses

We used the A/Aichi/2/68 (H3N2), A/Shanghai/2/2013(H7N9)-PR8-IDCDC, A/Chicken/Kurgan/05/05 RG (H5N1), A/California/07/09 H1N1pdm09, and A/California/1/66 (H2N2) influenza strains. The A/Aichi/2/68 (H3N2), A/California/1/66 (H2N2), and A/Kurgan/05/05 RG influenza strains were received from the Collection of Influenza and Acute Respiratory Viruses at the Research Institute of Influenza (RII). A/Kurgan/05/05 RG is an avirulent strain obtained by reverse genetics work at the RII. The virus was modified in order to remove the polybasic cleavage site in the viral hemagglutinin. The A/Shanghai/2/2013(H7N9)-PR8-IDCDC and California/07/09 H1N1pdm09 influenza strains were received from the Centers for Disease Control and Prevention (Atlanta, USA). The mouse-adapted variants were obtained at the Research Institute of Influenza by serial mouse/egg passages of source viruses. The mouse-adapted variants have retained the antigenic properties of the source viruses, yet have acquired the ability to lethally infect mice. Experimental work with the A/H2N2 and A/H5N1 influenza strains was carried in a BSL-3 facility.

### Serological analysis by enzyme-linked immunosorbent assays (ELISA)

Specific IgG and IgA titers were measured by enzyme linked immunosorbent assay (ELISA) using serum, BAL, or nasal samples from each mouse (n = 5), as previously described [37]. The plates were coated overnight at 4°C with the M2e peptides (5 μg/ml) or purified viruses (2 μg/ml) in PBS pH 7.2. The synthetic peptides used were M2es (SLLTEVE**T**PTR**S**EW**E**CRC**S**DSSD: M2e from A/California/09/07(H1N1)) or M2eh (SLLTEVETPIRNEWGCRCNDSSD: M2e from a consensus of human influenza A viruses). The purified viruses were A/Aichi/2/68(H3N2), A/California/07/09 (H1N1pdm09), A/California/1/66 (H2N2), A/Chicken/Kurgan/05/05 RG (H5N1), or A/Shanghai/2/2013(H7N9)-PR8-IDCDC H7N9. Polyclonal HRP-labeled goat anti-mouse IgG, IgG1, IgG2a, IgG2b, or IgG3 antibodies (Abcam, UK) were used. To detect IgA, polyclonal HRP-labeled goat anti-mouse IgA (Abcam, UK) was used. TMB (BD Bioscience, USA) was used as substrate; the incubation time was 15 min. The optical density (OD) was measured using an i-Mark microplate reader (Bio-Rad) at a wavelength of 450 nm. The maximal serum dilution that had an optical density at least double the mean value of the blank was taken as the titer.

### Intracellular cytokine staining (ICS) assay

Multi-parameter flow cytometry was performed in accordance with the BD Pharmingen^TM^ protocol. In brief, mouse lungs were harvested at day 14 post second boost; lung lymphocytes (5x10^6^) were stimulated in Nunc^TM^ 96-well conical bottom plates (Thermo Scientific™, USA) for 6 h at 37°C with 10 μg of M2eh peptide, in combination with 1 μg of either the A/Aichi/2/68 (H3N2) or the A/California/07/09 (H1N1pdm09) influenza virus, in the presence of 1 μg/ml of Brefeldin A (BD Bioscience, USA) and purified hamster anti-mouse CD28. The cells were then washed, and Fc receptors were blocked using CD16/CD32 antibodies (Mouse BD Fc Block, BD Pharmingen, USA) for 30 min. Next, cells were incubated with Zombie Aqua (Zombie Aqua™ Fixable Viability Kit, Biolegend, USA) to enable gating of live cells during analysis, and they were subsequently stained with CD3e-FITC, CD8a- APC-Cy™7, CD4- PerCP, CD62L-PE-Cy™7, or CD44-APC (BD Pharmingen, USA) antibodies at 4^0^ C for 30 min. Cells were permeabilized using the BD Cytofix/Cytoperm Plus (BD Bioscience, USA) protocols and stained with anti-TNF-α-BV421 or anti-IFN-γ-PE antibodies (BD Pharmingen, USA). Sample acquisition (50,000 live CD3+ T-cells were collected) was performed with a BD FACS Canto II flow cytometer (Becton Dickinson, USA) and analyzed using Kaluza version 1.5 (Beckman Coulter, USA).

### Influenza virus challenge

For viral challenge in mice, the A/California/1/66 (H2N2), A/Aichi/2/68 (H3N2), or A/Chicken/Kurgan/05/05 RG (H5N1) strains were used. Two weeks after the final immunization, the mice (n = 10/group) were anesthetized (2–3% isoflurane mixed with 30% oxygen and 70% nitrous oxide) and challenged i.n. with A/H3N2(10LD_50_), A/H2N2(10LD_50_), or A/H5N1 (5LD_50_) influenza A virus. Mice administered PBS were challenged as negative controls. The animals were monitored daily for survival and weight loss for 2 weeks. The collective weight of animals in a given group (experimental or control) were measured.

### Statistical analysis

Statistical data processing was carried out using the GraphPad Prism (version 6.0) program. The statistical significance of differences in antibody titers, and in antigen-specific cytokine-producing T-cells levels, were evaluated using the non-parametric Mann-Whitney U-test. The significance of differences in survival between mouse groups was analyzed by the Montel-Cox test. The differences were considered significant at the p< 0.05 level.

## Results

### *In silico* protein analysis

Several conserved epitopes of the influenza A virus, considered to be promising for the development of a recombinant vaccine, were used as follows: the ectodomain of the M2 protein (M2eh or M2es) and aa 76–130 of the hemagglutinin stalk. The aa 76–130 fragment comprises a long alpha-helix in the second subunit of HA which is partially accessible at the surface of the molecule. Alignment of viral subtypes AH3 and AH7 at the aa 76–130 region reveals a considerable degree of conservation: 63.6% (S1 Fig). When considering amino acid replacements by those with similar physical and chemical properties, the similarity is 80%. Protein sequence alignment of A/H1N1, A(H1N1)pdm09, A/H2N2, and A/H5N1 viral hemagglutinins in the same region shows 80% identity. When considering amino acid replacements by those with similar physical and chemical properties, the similarity is 90%. T- and B-cell epitopes of viral consensus sequences, restricted to the most prevalent HLA types in the human population, were analyzed and are represented in S3 and S4 Figs.

The first recombinant protein, Flg4M2e, contained four copies of M2e downstream of flagellin, arranged as M2eh- M2es- M2eh- M2es (**Fig 1A**). The second recombinant protein, termed Flg-HA2-2-4M2e, contained the 4M2e portion (mentioned above) and the HA2-2 fragment between flagellin and M2e. The third protein, Flg-HA2-1-4M2e, contained HA2-1 instead HA2-2.

**Fig 2:**
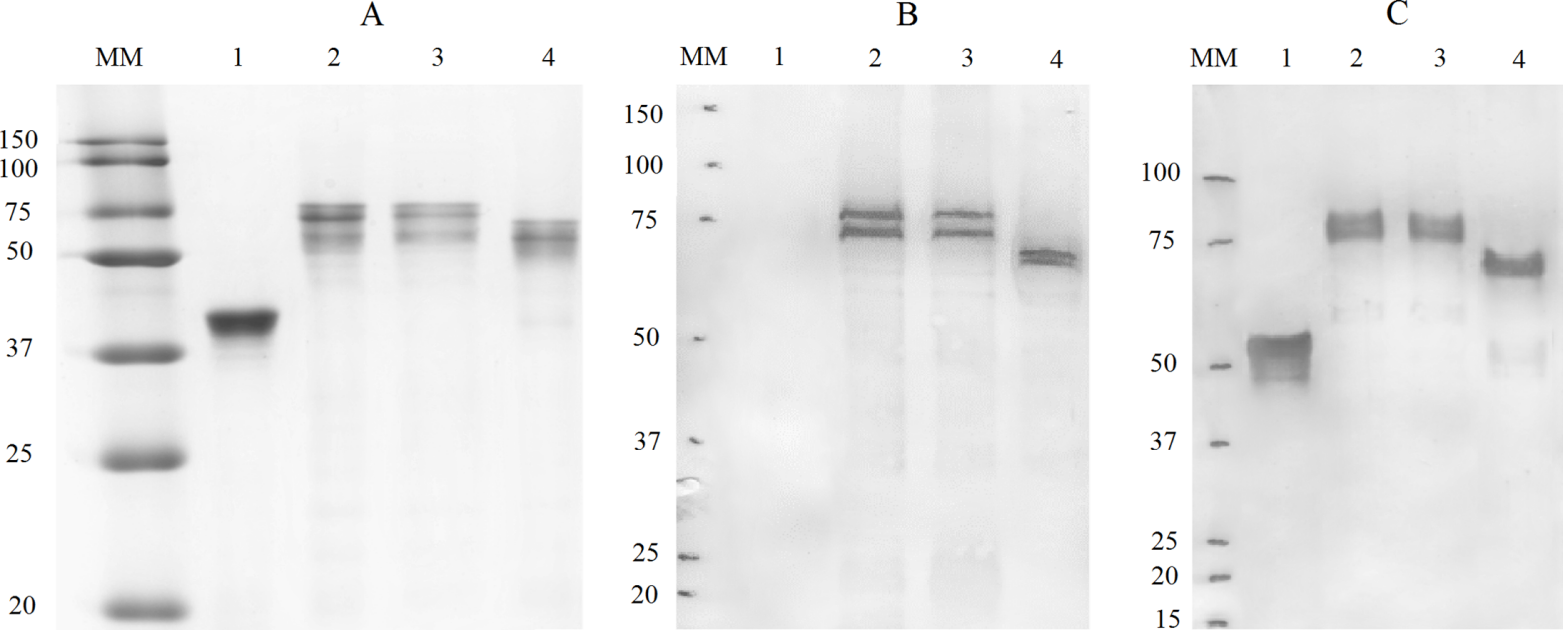
SDS-PAGE. **(A)** Coomassie brilliant blue staining. Western blot analysis of recombinant fusion proteins by (**B**) anti-M2e mAb 14C2, and (**C**) anti-Flg mAb 37913. The positions of molecular weight standards are indicated. 1: Flg-his; 2: Flg-H2-1-4M2e; 3: Flg-H2-2-4M2e; 4: Flg-4M2e.

Homology-based 3D modeling of structures in the Flg-HA2-2-4M2e and Flg-HA2-1- 4M2e recombinant proteins showed partial retention of the alpha-helix structure in the HA2 (76–130) fragment. The predicted folded structure of the recombinant featuring HA2-1 insert was more compact than the predicted structure of the construct featuring HA2-2 (**Fig 1B**). Such conformational differences in the structure of the two proteins may influence their immunogenicity. Recombinant fusion proteins were expressed in E. coli and purified. Expression levels reached 10–30% of total soluble protein. The theoretical molecular weights of the proteins coincided with their electrophoretic mobilities in SDS PAGE (**Fig 2A**).

The purified proteins were detected in an immunoblot using rabbit polyclonal antibodies against flagellin and an anti-M2e monoclonal antibody (mAb), 14C2. The blotting confirmed the protein’s identity and integrity (**Fig 2B and 2C)**. Given the fact that the 14C2 mAb has been shown to recognize protective epitopes in M2e [48], the current results demonstrate that the protective epitope of M2e is present and accessible in all of the recombinant proteins.

### Immunization with recombinant proteins induced specific anti-M2e and anti-HA antibodies in sera

**Fig 3:**
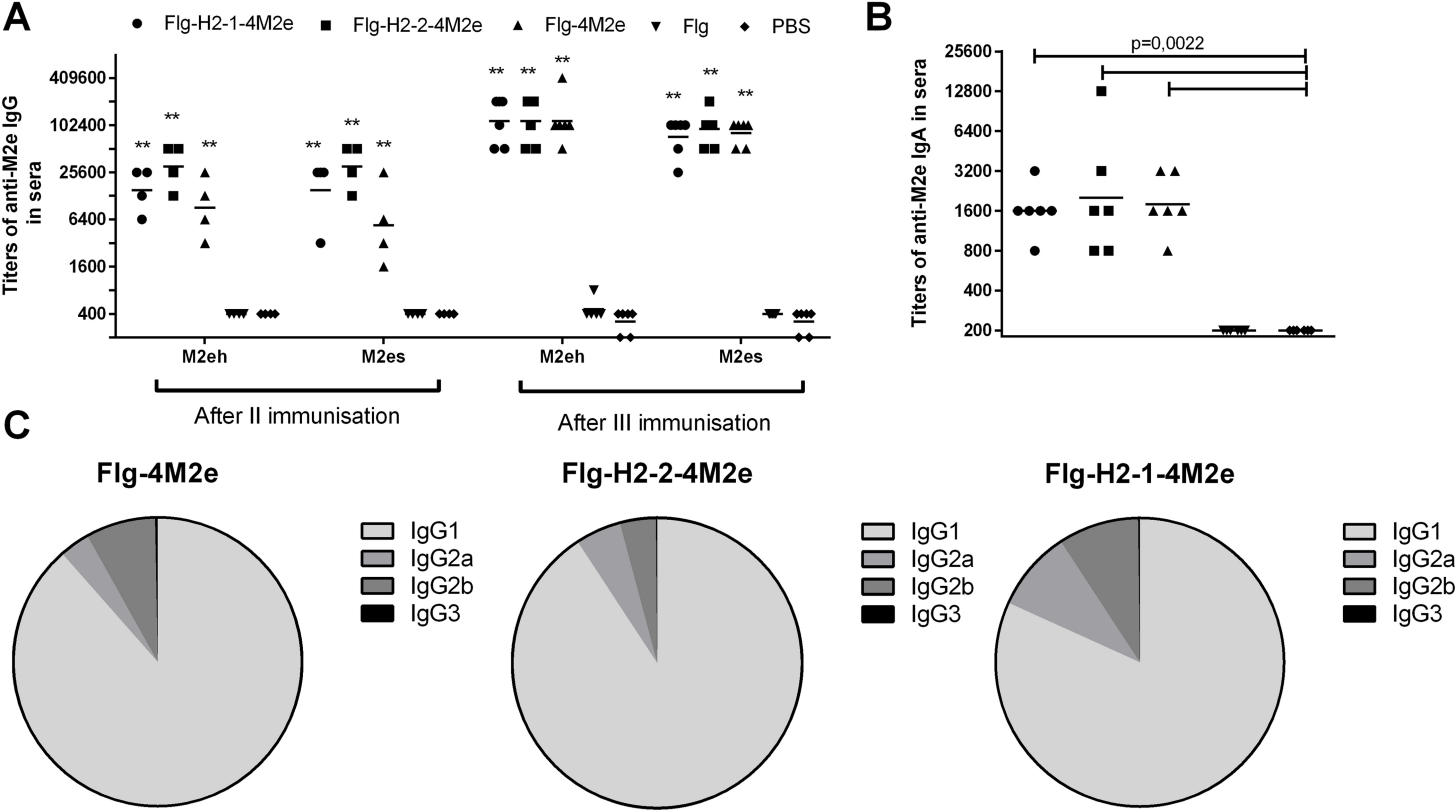
Anti-M2e antibody response in sera. BALB/c mice (n = 6/group) were immunized i.n. with 10 μg/0.02 ml of Flg-HA2-2-4M2e, Flg-HA2-1-4M2e, or Flg-4M2e on days 0, 14, 28. Mice in the control groups were administered with Flg-his (10 μg/0.02 ml) or PBS. Two weeks post second boost, **(A)** M2e-specific IgG (M2eh and M2es) and **(B)** IgA response in sera were evaluated by ELISA. (**C**) Anti-M2e IgG subclasses, tested against M2e peptide in sera, were determined by ELISA. Statistical significance was determined using the Mann-Whitney U-test. The P values between immunized and control group are indicated. **—significant difference from control groups, p<0.01.

In order to test the immunogenicity of the proteins (Flg-HA2-2-4M2e, Flg-HA2-1-4M2e, or Flg-4M2e), mice were immunized intranasally 3 times, with a 2 week break between immunizations. Sera were collected from mice 14 days after the last immunization and tested by ELISA for reactivity with the synthetic peptides M2eh, M2es, or with different subtypes of viruses. The levels of IgG1, IgG2a, IgG2b, and IgG3 subtypes, as well as IgA, were analyzed in sera. The immunogenicity of the Flg-HA2-2-4M2e and Flg-HA2-1-4M2e proteins was compared to that of the Flg-4M2e protein. According to ELISA data, all of the three proteins stimulated the production of high serum levels of anti-M2e IgG and IgA. The GMT range for IgG was 1:75,000–1:115,000 and 1:1,600–1:2,750 for IgA (**Fig 3A and 3B**).

In the case of IgG, high antibody titers were formed to both the M2eh and M2es peptides (**Fig 3A**). The specific anti-M2e IgG and IgA responses to immunization with the three positive experimental proteins showed no significant differences. We found that these proteins mostly induced antibodies from IgG1 subclass (**Fig 3C**). The IgG2a and IgG2b subclasses comprised from 9.0 to 18.0% of total IgG. These isotypes are of the greatest importance for animals with the FcɣR III (-/-) phenotype, whereas IgG1 isotype antibodies require functional FcɣR III to rescue mice from a lethal influenza virus challenge [49].

**Fig 4:**
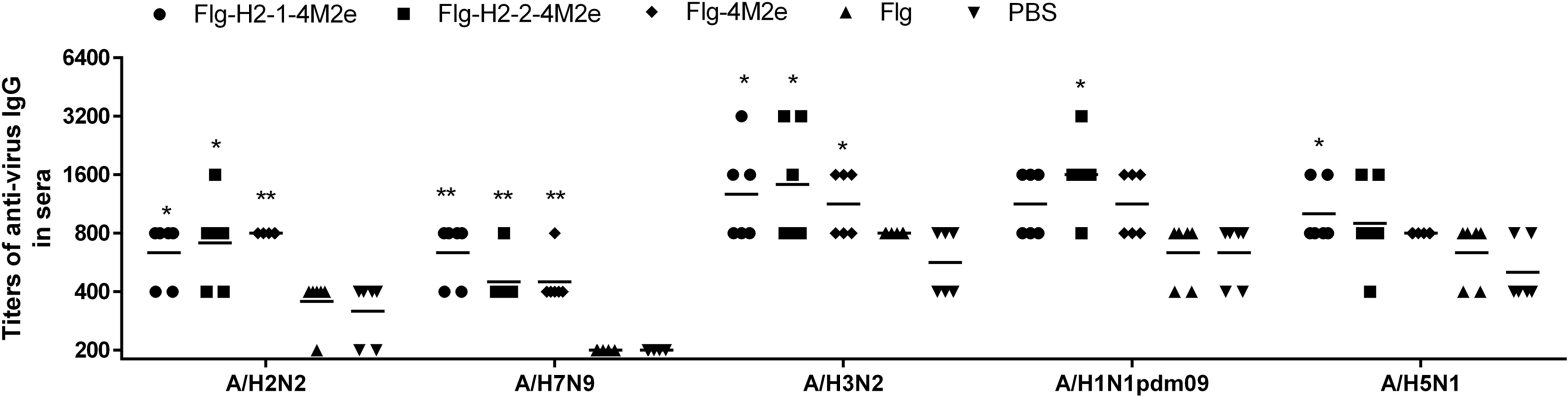
Anti-virus antibody response in sera. BALB/c mice (n = 6/group) were immunized i.n. with 10 μg/0.02 ml of Flg-HA2-2-4M2e, Flg-HA2-1-4M2e, or Flg-4M2e on days 0, 14, 28. Mice in the control group were administered with PBS. Two weeks post second boost, specific antibodies to purified viruses A/H2N2, A/H7N9, A/H3N2, A/H1N1pdm, and A/H5N1 were evaluated by ELISA. Statistical significance was determined using the Mann-Whitney U-test. The P values between immunized and control group are indicated. *—significant difference from control groups, p<0.05, **—significant difference from control groups, p<0.01.

Production of anti-HA antibodies to various subtypes of the influenza A virus was observed in sera as well. Although the production of these antibodies was modest and definitely weaker than the production of anti-M2e antibodies, they were specific to every one of the viral strains used in the study (**Fig 4**).

The antibody levels to viral subtypes H2, H1, H5, and H7 differed significantly from anti-HA antibody levels in control mice. Immunization with either of the proteins (Flg-HA2-2-4M2e or Flg-HA2-1-4M2e) led to the formation of specific antibodies to HA2 that were capable of binding with influenza viruses from both phylogenetic groups I and II. The GMTs of specific antibodies to viruses of different phylogenetic groups did not depend on which recombinant protein (Flg-HA2-2-4M2e or Flg-HA2-1-4M2e) was used for immunization.

### Candidate vaccines induced anti-M2e and anti-viral local IgA

Mucosal immune responses are important in the context of influenza infection because the virus invades via mucosal surfaces. In order to determine local, antigen-specific response patterns, mice were immunized i.n. with each one of the vaccine candidates, as described in the Materials and methods. The levels of local IgA, specific to M2e or purified virus (A/H2N2 or A/H7N9), in immunized animals were assessed by ELISA.

**Fig 5:**
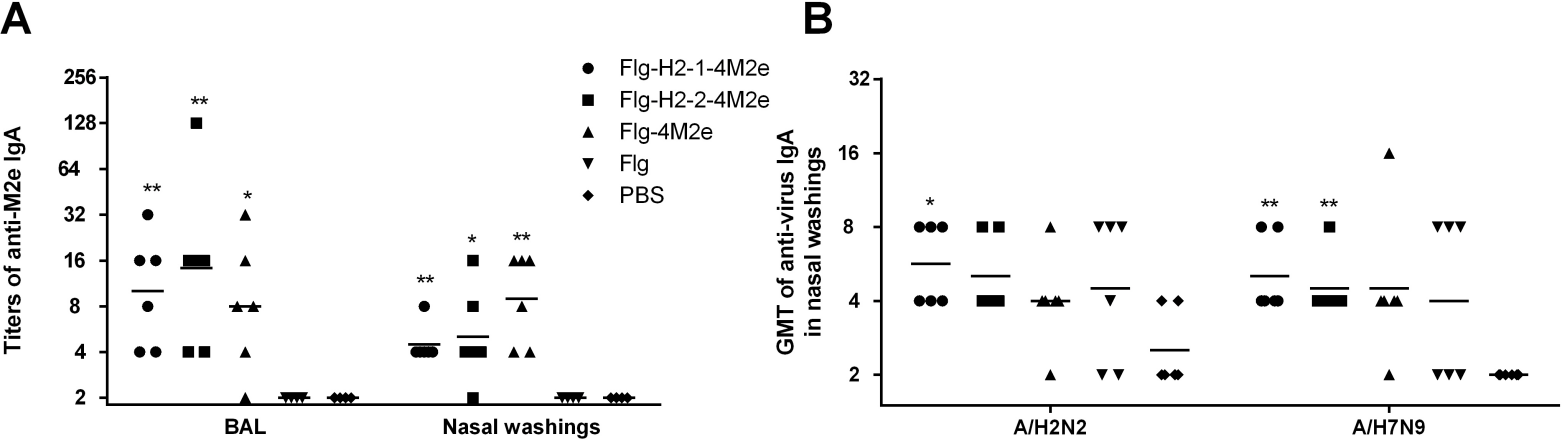
Antibody response in BAL and nasal washes. **(A)** Anti-M2e IgA in BAL and nasal washes. (**B)** Anti-virus (A/H2N2, A/H7N9) IgA in nasal washes. Nasal washes and BAL were collected and evaluated by ELISA two weeks after second boost. Statistical significance was determined using the Mann-Whitney U-test. The P values between immunized and control group are indicated. *- significant difference from control groups, p<0.05, ** -significant difference from control groups, p<0.01.

As shown in **Fig 5A**, all three of the vaccine formulations induced anti-M2e IgA, as measured in nasal washes, at comparable levels. Much like the serum antibody results, the mucosal anti-viral antibodies induced by the recombinants (Flg-HA2-2-4M2e or Flg-HA2-1-4M2e) did not significantly differ (**Fig 5B**). The titers of anti-M2e IgA in BAL were comparable after immunization with each of the three vaccine candidates, and they were 2-fold higher than the titers in nasal washes.

### Antigen-specific T cell response in lung

**Fig 6:**
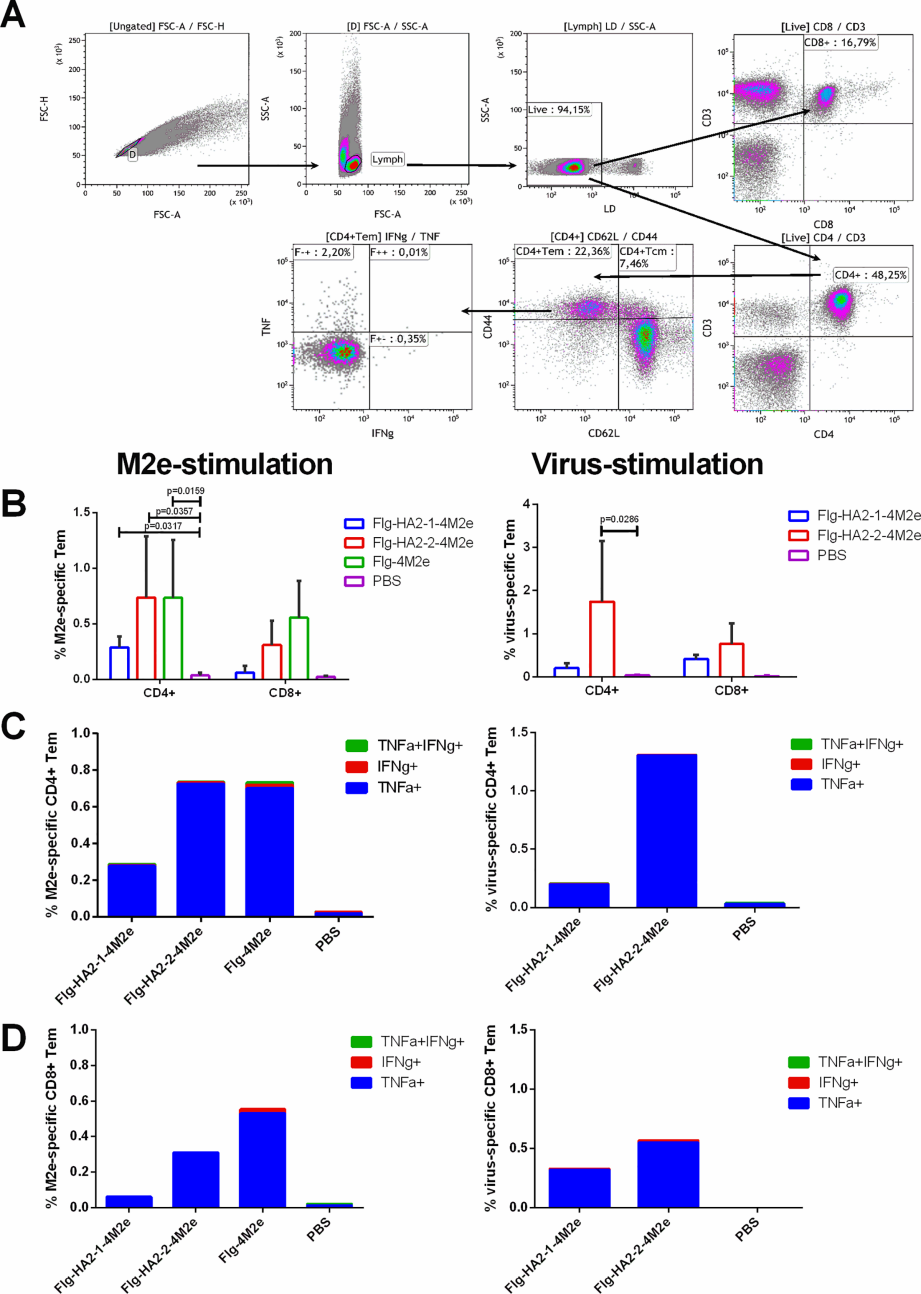
Antigen-specific CD4+ and CD8+ effector memory T-cells in lung. Lung lymphocytes were isolated from 5 mice of each group 2 weeks post final immunization and assayed for M2e-specific and virus-specific CD4+ and CD8+ T effector memory cells (Tem). (**A**) The gating strategy of single or double cytokine-secreting antigen-specific CD4+, CD8+ Tem. FSC-A/FSC-H: removal of cell doublets; FSC-A/SSC-A: gating of the lymphocyte population; LD/SSC-A: gating of the live lymphocyte population; CD8/CD3: gating of the live CD3+CD8+ lymphocyte population; CD4/CD3: gating of the live CD3+CD4+ lymphocyte population; CD62L/CD44: gating of the CD4+Tem and CD4+Tcm cell population; IFNg/TNF: gating of CD4+Tem cells (producing TNFa, IFNg or both). For the CD8+ cell population, the gating is similar. (**B**) Antigen-specific CD4+ and CD8+ Tem cells after re-stimulation with M2e or virus (A/H3N2 for mice immunized with FlgH2-2-4M2e; A/H1N1pdm09 for mice immunized with FlgH2-1-4M2e). (**C)** Antigen-specific cytokine-producing CD4+ Tem after re-stimulation with M2e and virus (A/H3N2 or A/H1N1pdm09). (**D)** Antigen-specific cytokine-producing CD8+ cells after re-stimulation with M2e and virus (A/H3N2 or A/H1N1pdm09). Data are presented as the mean±SEM. Statistical significance was determined using the Mann-Whitney U-test. The P values between groups are indicated.

To determine effector memory CD4+ and CD8+ T-cell responses (Tem, CD44+/CD62L-), lymphocytes were isolated from lungs of immunized and control mice (5 mice in each group) two weeks post second boost. They were re-stimulated with M2e peptide or influenza A virus (A/California/07/09(H1N1) or A/Aichi/2/68(H3N2)), and cytokine production was analyzed by intracellular cytokine staining assay. **Fig 6A** shows the gating strategy for single or double cytokine-secreting antigen-specific CD4+, CD8+ Tem cells.

An activation of T-cells in the control groups was not observed. Immunization with Flg-4M2e, as well as Flg-HA2-2-4M2e, induced a stronger anti-M2e immune response by CD4+ Tem and CD8+ Tem T-cells compared to Flg-HA2-1-4M2e (**Fig 6B**). The CD4+ Tem data for the recombinants (Flg-4M2e, Flg-HA2-2-4M2e, or Flg-HA2-1-4M2e) were 0.73%, 0.74%, and 0.29%, respectively. The M2e-specific CD8+ Tem data for the recombinants (Flg4-M2e, Flg-HA2-2-4M2e, or Flg-HA2-1-4M2e) were 0.55%, 0.31%, and 0.06%, respectively. M2e-specific CD4+ and CD8+ Tem TNF-producing cells were dominant, whereas IFN-producing T-cells were detected at a very low levels (**Fig 6C and 6D**). Lung cells, re-stimulated with the AH1N1 influenza virus (for mice immunized with Flg-HA2-1-4M2e) or the AH3N2 virus (for mice immunized with Flg-HA2-2-4M2e), displayed a similar pattern featuring a predominance of TNF-α producing CD4+ cells and much fewer CD8+ T cells (**Fig 6B, 6C and 6D**). It is worth noting that the recombinant protein with HA2-2 induced a greater T cell response (1.74% CD4+Tem and 0.76% CD8+Tem) than the protein with HA2-1 (0.2% CD4+Tem and 0.41% CD8+Tem). In general, the results demonstrate that immunization with the recombinant proteins, either Flg-HA2-2-4M2e or Flg-HA2-1-4M2e, stimulates a stronger immunogenic effect due to the T-cell response directed towards the second hemagglutinin subunit.

### Intranasal immunization of mice with the recombinant proteins protects against lethal heterologous challenge

**Fig 7:**
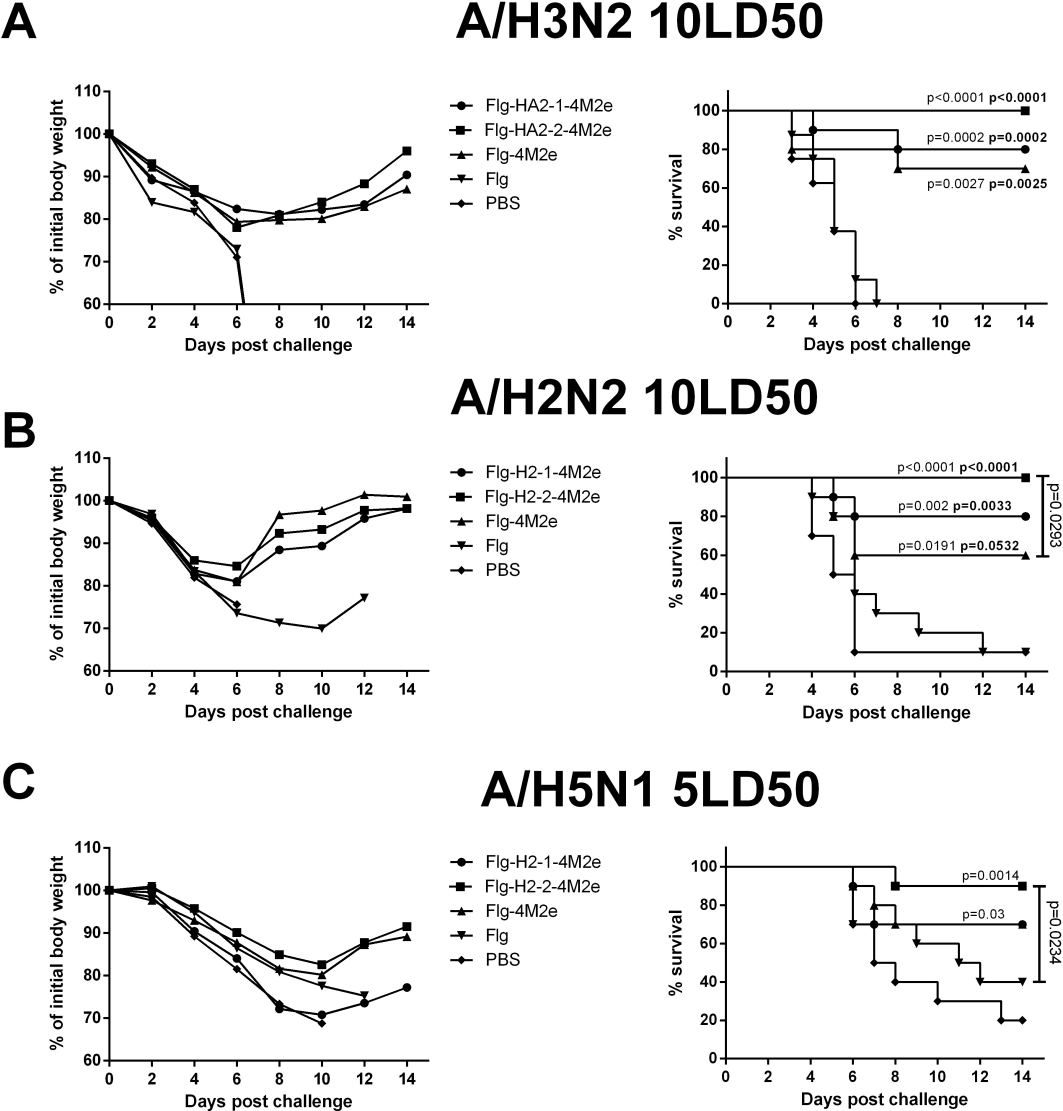
Efficacy of immunization. Groups of 10 Balb/c mice were immunized with Flg-HA2-2-4M2e, Flg-HA2-1-4M2e, or Flg-4M2e fusion proteins (10 μg/0.02 ml). Control group mice were administered Flg-his (10 μg/0.02 ml) or PBS. Two weeks post-second boost, mice were challenged with (**A**) 10LD50 A/Aichi/2/68 (H3N2), (**B**) 10LD50 A/ California/1/66 (H2N2), or **(C)** 5LD50 A/Kurgan/05/05 RG (H5N1) virus. Body weight (left) and survival rate (right) were monitored daily for 14 days. The collective weight of animals in a given group (experimental or control) was determined daily for 2 weeks. The P values (Montel-Cox test) between immunized and control groups, and between groups immunized with different proteins, are indicated (regular font: difference from PBS group; bold font: difference from Flg-his group).

Two weeks after the second boost, mice were challenged with lethal doses of mouse-adapted viruses, namely: AH3N2 (10LD_50_), AH2N2 (10LD_50_), or AH5N1 (5LD_50_). Body weight and survival were monitored for 14 days. Immunization with each of the recombinant proteins provided significant protection; mouse survival rates were 60–100% (**Fig 7**).

However, a stronger protective effect was obtained after immunization with recombinant proteins containing both the M2e and HA2 (aa76-130) viral peptide fragments. When challenged with viruses belonging to both phylogenetic groups, mice immunized with Flg-HA2-2-4M2e or Flg-HA2-1-4M2e survived in 70–100% of the cases, whereas mice immunized with Flag4M2e survived in 60–70%. When the two constructs containing an HA2 segment (Flg-HA2-2-4M2e and Flg-HA2-1-4M2e) were compared, stronger results were obtained with the HA2-2-containing peptide. Immunization of mice with Flg-HA2-2-4M2e provided complete protection against lethal challenge with the A/Aichi/2/68 (H3N2) and A/California/1/66 (H2N2) influenza viruses. In 90% of cases, it also provided protection against challenge with the A/Chicken/Kurgan/05/05 RG (H5N1) virus. Flg-HA2-1-4M2e, on the other hand, provided a survival rate of 80% after challenge with the A/H2N2 virus and a survival rate of 70% after challenge with the A/H5N1 virus. Mice that received the Flg-HA2-2-4M2e protein lost considerably less weight, than did mice immunized with either the Flg-HA2-1-4M2e or Flg-4M2e proteins. The weight losses measured were: 15–18% (Flg-HA2-2-4M2e group); 19–30% (Flg-HA2-1-4M2e group); and 20–30% (Flg-4M2e group) (**Fig 7**).

## Discussion

In this study, we analyzed three recombinant proteins in terms of their immunogenicity and their abilities to elicit protective responses. The responses induced by the Flg-4M2e protein were surpassed by superior responses induced by Flg-HA2-1-4M2e and, especially, Flg-HA2-2-4M2e. The Flg-4M2e protein includes four M2e molecules; two molecules are a consensus sequence of human influenza A viruses and two M2e molecules are from the A(H1N1)pdm09 virus. Their sequences differ from each other by 4 amino acids, including one which is critical for binding antibodies, position eleven [50]. The Flg-HA2-2-4M2e and Flg-HA2-1-4M2e proteins are identical with respect to their copies of M2e, but differ according to which HA2 region (aa 76–130) was used. The HA2 from phylogenetic group II (HA2-2) represents a consensus from the A/H3N2 and A/H7N9 viruses. The HA2 from phylogenetic group I (HA2-1) represents a consensus from the A/H1N1, A/H1N1pdm, A/H2N2, and A/H5N1 viruses. The decision to use HA2 consensus derived from H3 and H7 viral subtypes was due their epidemiological significance. The A/H3N2 virus has been circulating in the human population since 1968, and it causes almost yearly influenza epidemics. The A/H7N9 virus has caused 5 epizootics in China since February 2013 alone [51, 52]. It is considered as a possible gene donor for future pandemic viruses. The viruses which were selected from the first phylogenetic group are either circulating now in the population as A/H1N1pdm, or pose a threat as potential pandemic viruses (A/H2N2, A/H5N1). The recombinant proteins produced, including their conserved antigen sequence designs, were fused genetically with bacterial flagellin, which is an effective mucosal adjuvant.

The cross-protective properties of M2e-based vaccines have been proven by numerous studies [32, 34, 37, 53]. Some studies have demonstrated the efficacy of polypeptides matching positions aa76-130 or aa24-184 in the hemagglutinin second subunit as target proteins in various vaccine candidates [6, 27, 54, 55]. The rationale for including HA2 was to increase the number of targets for effectors of the immune system. Some neutralizing epitopes are known to be located in the HA2 region [21, 55]. One of them is located in the long alpha helix and binds the 12D1 mAb [21].

In general, the immune response to the M2e and HA2 (aa76-130) peptides is directed at different stages of the viral infection in a host. The anti-M2e immune response is directed mainly at infected cells. The antibody-M2e complex on the infected cell surface is recognized by Fcɣ receptors on macrophages and other types of cells; this interaction may promote antibody-depend cytotoxicity or phagocytosis, thereby preventing the spread of viral infection [56, 57].

Anti-HA-2 specific antibodies employ various mechanisms of direct and indirect neutralization. Antibodies directed at epitopes of the long alpha helix inhibit pH-induced conformational changes of HA within endosomes [17, 21, 24]. This mechanism prevents fusion of the viral and endosomal membranes, and it prevents release of genomic material into the cytosol. Broadly neutralizing antibodies may also inhibit viral egress and HA maturation by steric hindrance of the interaction between host proteases and the cleavage site. At a minimum, these antibodies were capable of mediating cytotoxicity of infected cells through Fc-FcɣR interaction [16, 26]. A number of authors have shown the cross-protective efficacy of HA2 fragments (aa 76–130) [6, 27].

We have shown that the introduction of the aa 76–130 polypeptide into the recombinant Flg4M2e protein led to the formation of anti-viral IgG antibodies, in addition to the anti-M2e IgG antibodies induced by the simpler Flg-4M2e protein. Both of the vaccine candidates (Flg-HA2-2-4M2e and Flg-HA2-1-4M2e) were cross-reactive and induced the production of antibodies against viruses from different phylogenetic groups. At the same time, neutralization reaction with these antibodies did not yield positive results (data not shown). It has been determined that neutralizing Abs directed against viruses of both phylogenetic groups interact with conformational epitopes, including residues of the alpha-helix, the large portion of fusion-peptide, and the HA0 cleavage site [22, 24, 58]. In our recombinant proteins, alpha-helix sequences were included, however this motif was apparently insufficient to stimulate neutralizing antibodies.

Some data indicate that HA stalk-binding antibodies perform optimally when in a polyclonal context and that the induction of HA stalk–specific IgA antibodies should be an important consideration during universal influenza vaccine design [59]. Detailed analysis of the biological activity of stalk-binding IgA have demonstrated an enhancement in IgA-mediated HA stalk neutralization, relative to that achieved by antibodies of IgG isotypes. In some cases, the neutralizing activity of IgA was equivalent to that of IgG [59]. Our results have shown that the Flg-HA2-2-4M2e and Flg-HA2-1-4M2e recombinant proteins induced the production of anti-M2e and anti-virus antibodies (both IgG as well as IgA) in sera.

All of the recombinants used in the study were able to induce a considerable immune response, as measured both in nasal washes and BAL. The formation of local immunity is one of the advantages of intranasal vaccine administration [60]. It has been shown that i.n. administration of M2e -based vaccine induced stronger protection against influenza virus than parenteral immunization [61]. Furthermore, several studies have demonstrated a significant role of mucosal IgA in cross-protection [62, 63] and in reducing viral transmission [64]. Although the titers of anti-viral antibodies, in both serum and nasal washes, were lower than the titers of anti-M2e antibodies, their contribution to the protective effect of the vaccine was real.

T-cells play an important role in anti-viral cross-protective immunity. Broadly protective immunity against influenza can be provided by memory CD4 and CD8 T-cells [65]. The role of tissue-resident lung memory CD4+ T cells is particularly important [65, 66]. Effector memory CD4+ T-cells simultaneously act through multiple pathways to provide a high level of protection: they provide helper functions to B and CD8 T-cells; they regulate innate immunity; and they can directly combat pathogens [67]. Our results show that intranasal immunization of mice with the Flg-HA2-2-4M2e or Flg-4M2e proteins stimulated effector memory M2e-specific CD4+ T-cells in lung (predominantly single-producers of TNF-α) in comparable quantities, while immunization with Flg-HA2-1-4M2e stimulated M2e-specific memory CD4+ T-cells in a much lesser quantity. The target HA2 sequences from influenza phylogenetic group II demonstrated a noticeable formation of HA2-specific effector memory CD4+ T-cells in lung. That cell pool may potentially go on to form a population of TNF-α-producing CD4+ cells. The latter can substantially influence antiviral CD8+ T-cell response and is essential for attenuating peak CD8+ T-cell responses following acute influenza virus infection [68]. However, the induction of that important aspect of the response, namely IFNγ–producing T cells, was insignificant. Only low or undetectable levels of specific CD8+ were seen in response to antigen re-stimulation. According to our unpublished data, the insertion of HA2-2 (aa76-130) into the hypervariable domain of flagellin greatly increases IFNγ–producing T-cells and shifts the IgG profile in the direction of increased IgG2a and IgG2b.

We have demonstrated that a stronger T-cell immune response and a more robust protective effect were obtained after immunization with recombinant protein containing the HA2-2 fragment. When challenged with a 10LD_50_ load of viruses belonging to both phylogenetic groups, immunized animals demonstrated a strong immune response which kept them alive in 90–100% of cases. The Flg-HA2-l-4M2e and Flg-4M2e proteins protected only 75% of animals. The weaker immunogenicity and protection provided by the FlgHA2-1-4M2e protein is probably due to conformational differences which affect the accessibility of the epitopes to CD4+ and B cells. These epitopes in the 76–130 region of HA2-2 are more dispersed, whereas in the same region of HA2-1, many of the epitopes overlap each other.

## Conclusion

The novelty of this investigation lays in the original recombinant protein designs which combine flagellin (a ligand of TLR5) as a carrier with conserved M2e and influenza A HA2 peptide sequences. These protein constructs (Flg-HA2-2-4M2e and Flg-HA2-1-4M2e) are highly immunogenic, and they stimulate both mucosal and systemic immune responses, including M2e-specific and virus-specific lung-resident effector memory T-cells. Both vaccine candidates were cross-reactive and induced the production of antiviral antibodies against viruses from different phylogenetic groups. However, the immunogenicity and protective efficacy of the target HA2 consensus peptide, derived from H3 and H7 viral hemagglutinin variants, were better than those resulting from immunization with recombinants using a phylogenetic group I HA2 consensus fragment. The protein comprising both M2e and HA2-2 should be considered for future development as a universal vaccine candidate.

## Supporting information

S1 ARRIVE Checklist ARRIVE guidelines checklist. (DOCX)

S1 Fig Amino acid sequence alignment of influenza A virus hemagglutinin from phylogenetic group I. Consensus H1: seasonal human influenza A/H1N1 viruses. Consensus H1pdm: pandemic human influenza A/H1N1pdm viruses. Consensus H2: human and bird influenza viruses of the subtype H2N2. Consensus H5: avian influenza viruses, including those isolated from humans, subtype H5N1. The start of the HA2 subunit is indicated by the arrow; sequences of HA2(76–130) are underlined in red; identical sequences are shown in yellow; substitutions by amino acids similar in properties are shown in green; amino acid substitutions are marked no color; insertions are shown in blue.
(DOCX)

S2 Fig Amino acid sequence alignment of influenza A virus hemagglutinin from phylogenetic group II. Consensus H3: human influenza viruses A/H3N2. Consensus H7: influenza viruses of subtype H7, including those isolated from humans. The start of the HA2 subunit is indicated by the arrow; sequences of HA2 (76–130) are underlined in red; identical sequences are shown in yellow; substitutions by amino acids similar in properties are shown in green; amino acid substitutions are marked no color; insertions are shown in blue.
(DOCX)

S3 Fig Experimental B- and CD4+ T-cell epitopes. Result of IEDB database search is presented for sequence of influenza viruses from phylogenetic groups I (**A**) and II (**B**). Non-homologous amino acids are marked with red. Green font identifies the single B-cell epitope. Black font identifies the CD4+ T-cells epitopes.
(TIF)

S4 Fig Potential CD8+ T-cell epitopes inside the HA2 (aa76-130) fragment for a representative set of alleles; results of analysis using NetCTLpan1.1 server are shown. Blue font identifies the CD8+ T-cells epitopes.
(TIF)

## Abbreviations

BSA: bovine serum albumin
CTL: cytotoxic T-lymphocyte
ELISA: enzyme-linked immunosorbent assay
HA: hemagglutinin
HA1: first subunit of hemagglutinin
HA2: second subunit of hemagglutinin
ICS: intracellular cytokine staining
i.n.: intranasal immunization
LD50: 50% lethal dose
MID: mouse infectious dose
MDCK: Madin-Darby canine kidney
M2e: ectodomain of M2 protein
OD: optical density
PBS: phosphate buffer solution
PCR: polymerase chain reaction
SDS-PAGE: sodium dodecyl sulfate polyacrylamide gel electrophoresis
s.c.: subcutaneous immunization
TLR5: Toll-like receptor 5
TMB: 3,3', 5,5' tetramethylbenzidine
Tem: effector memory T-cell
Tcm: central memory T-cell
