# Supplementary material for: Combination of M2e peptide with stalk HA epitopes of influenza A virus enhances protective properties of recombinant vaccine
Source: PLoS One. 2018 Aug 23;13(8):e0201429. doi: 10.1371/journal.pone.0201429 (PMC6107133; doi:10.1371/journal.pone.0201429)
Supplement: S1 ARRIVE Checklist — (DOCX) [file pone.0201429.s001.docx]

**S1 Checklist. ARRIVE Guidelines Checklist.**

Liudmila M. Tsybalova^1^*, Liudmila A. Stepanova^1^, Marina A. Shuklina^1^, Eugenia S.Mardanova^2^, Roman Y. Kotlyarov ^2^, Marina V. Potapchuk^1^, Sergei A.Petrov^1^, Elena A. Blokhina ^2^, Nikolai V. Ravin^2^

|  | Item |  |
| --- | --- | --- |
| Title | 1 | Combination of M2e peptide with stalk HA epitopes of influenza A virus enhances protective properties of recombinant vaccine |
| Abstract | 2 | BACKGROUND AND PURPOSE: Influenza infection could be more effectively controlled if a multi-purpose vaccine with the ability to induce responses against most, or all influenza A subtypes could be generated. Conserved viral proteins are a promising basis for the creation of a broadly protective vaccine. In the present study, the immunogenicity and protective properties of three recombinant proteins (vaccine candidates), comprising conserved viral proteins fused with flagellin, were compared.  EXPERIMENTAL APPROACH**:** Balb/c mice were immunized intranazally on the days 0 (primary), 14 (first boost), 28 (second boost) with 10 μg/0.02ml with recombinant proteins comprising either one viral protein (the ectodomain of the M2 protein, ‘M2e’) or two viral proteins (M2e and polypeptide aa 76-130 of hemagglutinin second subunit - HA2) genetically fused with flagellin. Further, two different consensus variants of HA2 were used. Therefore, three experimental positives were used in addition to the negative control (Flg-his or PBS). On day 42 we determined anti-M2e IgG, IgG isotypes, IgA in blood and IgG, IgA in BAL and nasal wash. At day 14 post immunization CD4+ and CD8+Tem-cells in lungs activated with M2e peptide and influenza viruses were tested on production of TNF-α and IFN-γ by Intracellular Cytokine Staining (ICS) assay. Mice were challenged intranazally 2 weeks after the final immunization with lethal doses of influenza viruses A/H2N2), A/H3N2, A/H5N1. The animals were monitored daily during 2 weeks for survival, and weight loss.  KEY RESULTS: We have demonstrated that insertion of the HA2 polypeptide (aa 76-130) consensus, derived from either the first (HA2-1) or second (HA2-2) virus phylogenetic groups, into the recombinant Flg4M2e protein significantly enhanced its immunogenicity and protective properties. Intranasal administration of the vaccine candidates (FlgHA2-2-4M2e and FlgHA2-1-4M2e) induced considerable mucosal and systemic responses including both anti-M2e and anti-virus activities. However, the immune response elicited by the FlgHA2-1-4M2e protein was weaker than the one generated by FlgHA2-2-4M2e. These recombinant proteins containing both viral peptides provide complete protection from lethal challenge with various influenza viruses: A/H3N2; A/H2N2; and H5N1.  CONCLUSION AND IMPLUCATIONS: This study demonstrates that the intranasal administration of FlgHA2-2-4M2e recombinant protein induces a strong immune response which provides broad protection against various influenza viruses. This protein is a promising candidate for the development of a universal vaccine. |
| INTRODUCTION | | |
| Background | 3 a | Conserved surface-exposed antigens, such as the extracellular domain of the M2 protein (M2e) and the stalk region of hemagglutinin (HA2), are the most promising targets for vaccine formulations that can protect against multiple influenza A virus subtypes [Kople A et al, 2017, Wang T.T et al, 2010]. Bacterial flagellin, as a natural ligand of Toll-like receptor 5 (TLR5), is a prospective adjuvant and a carrier for vaccine proteins [Le Moigne V et al 2008]. It is able to facilitate an effective immune response, even to poorly immunogenic epitopes, such as the influenza virus M2e peptide. One of the advantages of flagellin is its efficiency as an adjuvant for intranasal administration. In this study, we enhanced the protective effect of the vaccine candidate by including in the flagellin fusion protein both the M2e peptide and the fragment of HA stalk domain (aa 76-130). |
|  | b | For this study we used Balb/c mice as a common object for research of immune response to vaccines and protection against influenza A viruses. |
| Objectives | 4 | The objective of this work was to compare the immunogenic properties and the protective effect of candidate vaccines on the basis recombinant proteins, including two (M2e and aa76-130 of HA2) or one (M2e) influenza A virus protein. |
| Ethical statement | 5 | The study was carried out in strict accordance with Russian Guidelines for the Care and Use of Laboratory Animals (1977) and Committee on the Ethics of Animal Experiments of Research Institute of Influenza (Protocol Number: 1701). All efforts were made to minimize the suffering of the animals. Mice were housed in cages provisioned with water and standard food and monitored daily for health and condition. More than 30% body weight loss was used as a criterion for early euthanasia. The animals were euthanized by CO2 inhalation for 5 minutes. After final monitoring (14 day post challenge) all the survived mice were humanely euthanized using CO2 inhalation for 5 minutes. |
| Study design | 6 a | Four groups of 30 mice and one of 20 each were studied in protection experiments (lethal dose challenge). All mice were immunized intranasally with different candidate vaccines:  1.1 Female BALB/c mice immunized with FlgHA2-2-4M2e and challenged with A/Chiken/Kurgan/05/05 RG (H5N1); 1.2 Female BALB/c mice immunized with FlgHA2-2-4M2e and challenged with A/California/1/66 (H2N2); 1.3 Female BALB/c mice immunized with Flg- FlgHA2-2-4M2e and challenged with A/Aichi2/68 (H3N2);  2.1 Female BALB/c mice immunized with FlgHA2-1-4M2e and challenged with A/Chiken/Kurgan/05/05 RG (H5N1); 2.2 Female BALB/c mice immunized with FlgHA2-1-4M2e and challenged with A/California/1/66 (H2N2);  3.1 Female BALB/c mice immunized with Flg-4M2e and challenged with A/Chiken/Kurgan/05/05 RG (H5N1); 3.2 Female BALB/c mice immunized with Flg-4M2e and challenged with A/California/1/66 (H2N2); 3.3 Female BALB/c mice immunized with Flg-4M2e and challenged with A/Aichi2/68 (H3N2);  4.1 Female BALB/c mice immunized with Flg-his and challenged with A/Chiken/Kurgan/05/05 RG (H5N1); 4.2.Female BALB/c mice immunized with Flg-his FlgHA2-2-4M2e and challenged with A/California/1/66 (H2N2); 4.3 Female BALB/c mice immunized with Flg-his and challenged with A/Aichi2/68 (H3N2);  5.1 Female BALB/c mice immunized with PBS and challenged with A/Chiken/Kurgan/05/05 RG (H5N1);  5.2 Female BALB/c mice immunized with PBS and challenged with A/California/1/66 (H2N2); 5.3 Female BALB/c mice immunized with PBS and challenged with A/Aichi2/68 (H3N2).  Five groups of 6 mice each were studied in immune response experiments. |
|  | b | In all experiments in this study animals were randomized into immunized and control groups without individual marking |
|  | c | In the study, n refers to number of animals, number of samples from each experimental group or individual experimental group for mean body weight estimation. |
|  | d | Body weight, survival rate  priming  second boost  challenge  first boost  Days -7_ __0_ 1___________14____________28____________42_________________56_  - blood sampling, - BAL sampling, - lung sampling,    Figure 1. Experimental timelines. Mice were immunized on day 0 (priming), 14 (first boost), 28 (second boost). Two weeks post second boost (42 day) mice were challenge with lethal dose of influenza viruses. The animals were monitored daily during 2 weeks for survival and weight loss. |
| Experimental procedures | 7 a | All mice were immunized and challenged intranasally (0.02 ml of preparation in two nostrils) under inhalation anesthesia (isoflurane 2-3% mixed with 30% oxygen (O2) and 70% nitrous oxide (N2O)). Mice were sacrificed by CO_2_-box for euthanasia for 5 minutes (Vet Tech Solutions) for blood, BAL, lung sampling. More than 30% body weight loss was used as a criterion for early euthanasia. After final monitoring (14 day post challenge) all the survived mice were humanely euthanized using CO2 inhalation for 5 minutes. |
|  | b | All manipulations with animals (immunization, challenge, sampling) were performed in the light phase (morning) |
|  | c | The animals were tested in home cage |
|  | d | The intranasal route of immunization may be used for the recombinant proteins on the basis of flagellin because flagellin is an effective mucosal adjuvant. |
| Experimental animals | 8 a | Female BALB/c (17.4±1.6), aged 6-8 weeks, were included (n=170). |
|  | b | Female BALB/c mice were purchased from the “Stolbovaya” (Moscow region, Russia) and acclimatized for 7 days. Vendor health reports indicated that mice were free of known viral, bacterial, parasitic pathogens and influenza virus naïve. |
| Housing and husbandry | 9 a | Animals were housed in type 2 cages (10 mice in cage) filled with Lignocel (hygiene animal bedding). |
|  | b | Animals were housed in a 12-houre light/dark cycle, with room temperature (22±1^0^C) and humidity (55±5%) controlled room. All mice were allowed free access to water and maintenance diet containing 1.1% calcium (Chara diet, Assortment agro, Russia). |
|  | c | All animals were monitored daily during experiment for health status. |
| Sample size | 10 a | One hundred and seventy mice were used in experiment. Ninety eight animals of groups 1.1-1.3, 2.1-2.2, 3.1-3.3 were immunized intranasally (i.n.) with Flg-HA2-2-4M2e (36 mice) or Flg-HA2-1-4M2e (26 mice) or Flg-4M2e (36 mice). Seventy two animals of groups 4.1-4.3 (36 mice) and 5.1-5.3 (36 animals) received intranasally with Flg-his or PBS respectively and served as control. |
|  | b | The difference between antibody levels (n=6/group), percent of cytokine-secreting antigen-specific CD4^+^ and CD8^+^ Tem cells (n=5/group) was evaluated by Mann-Whitney U-test. Significant differences in survival among mouse groups (n=10/group) were analyzed by Montel-Cox test. |
|  | c | - |
| Allocating animals to experimental groups | 11 a | Mice were allocated in research and control groups in a random way, thus ensuring the mean body weight of mice in each group was similar prior to beginning of experiment. |
|  | b | For the protection experiment animals for research and control group were selected in a random way. For the anti-M2e and anti-virus immune response experiment animals from immunized and control groups were selected in a random way. |
| Experimental outcomes | 12 | One primary outcome result was analyzed: titers of anti-M2e and anti-virus IgG (day 42). In addition, two secondary outcome measures were evaluated: anti-M2e (IgG1, IgG2a, IgA) humoral and T-cell immune response (day 42), protection of immunized mice from lethal viral challenge (days 42-56). |
| Statistical methods | 13 a | Mann-Whitney U-tests for non-normally distributed data (ELISA results, T-cell immune response results) were performed to compare the differences in immunized and control mice. Montel-Cox test was performed to compare the difference in survival rate between immunized and control mice. |
|  | b | The experimental unit was an individual mice or sample (blood, BAL, lung) from individual mice or individual experimental group for mean body weight estimation. |
|  | c | - |
| RESULTS | | |
| Baseline data | 14 | The animals’ health status was monitored by a health surveillance programme according to Recommendation of the European Laboratory Animal Science Associations 2001 (FELASA) “Recommendation for health monitoring of rodent and rabbit colonies in breeding and experimental units”. |
| Number analysed | 15 a | Antibody response was assessed for 6 mice, T-cells response were assessed for 5 mice in each from 5 groups: 1, 2, 3, 4, 5. Eighteen mice from immunized group (18/20) and zero from control (0/20) survived post challenge with А/Aichi/2/68 (H3N2). Twenty four mice from immunized group (24/30) and two from control (2/20) survived post challenge with А/California/1/66 (H2N2). Twenty three mice from immunized group (23/30) and six from control (6/20) survived post challenge with А/Kurgan/5/05 RG (H5N1). |
|  | b | One hundred and seventy animals were utilized for this study and 170 were included and completed. |
| Outcomes and estimation | 16 | In accordance with ARRIVE guidelines (Kilkenny et al. 2010) we have reported measures of precision, confidence, and n to provide an indication of significance.  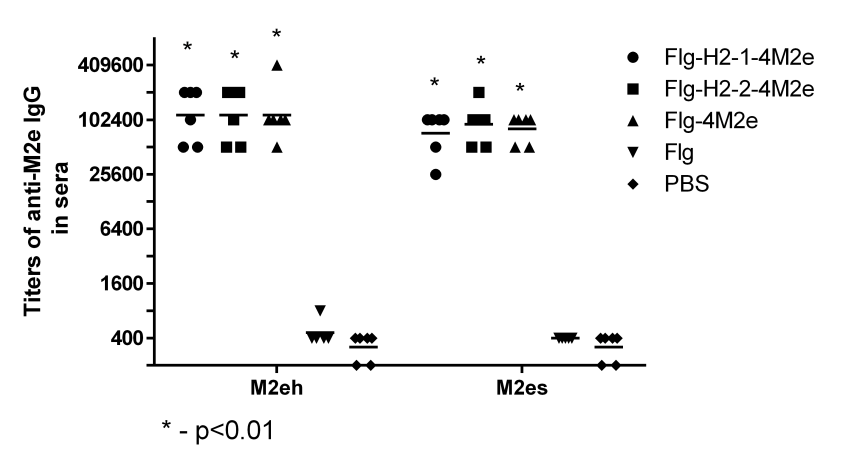  **Fig 3 Anti-M2e antibodies response in sera**. BALB/c mice (n = 6/group) were immunized i/n with 10 μg/0.02 ml of Flg-HA2-2-4M2e, Flg-HA2-1-4M2e or Flg-4M2e on days 0, 14, 28. Mice of the control groups were administered with Flg-his (10 μg/0.02 ml) or PBS. Two weeks post second boosting M2e-specific IgG (M2eh and M2es) were evaluated by ELISA. Statistical significance was determined using Mann-Whitney U-test. The P values between immunized and control group are indicated.  * - significant difference from control groups, p<0.01.  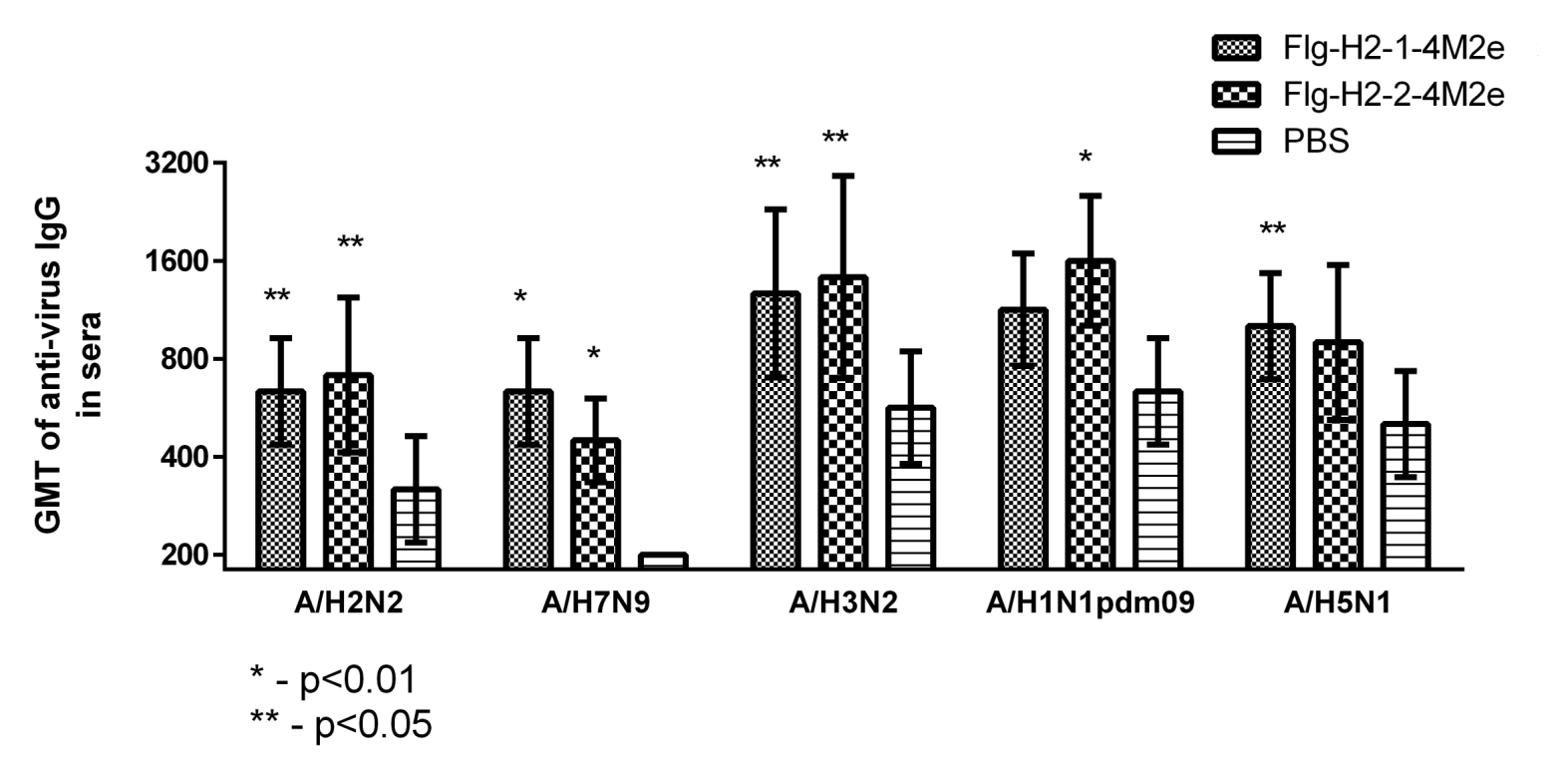  **Fig 4. Anti-viruses antibodies response in sera**. BALB/c mice (n = 6/group) were immunized i/n with 10 μg/0.02 ml of Flg-HA2-2-4M2e, Flg-HA2-1-4M2e or Flg-4M2e on days 0, 14, 28. Mice of control group were administered with PBS. Two weeks post second boosting specific antibodies to purified viruses A/H2N2, A/H7N9, A/H3N2, A/H1N1pdm and A/H5N1 were evaluated by ELISA. Statistical significance was determined using Mann-Whitney U-test. The P values between immunized and control group are indicated. * significant difference from control groups, p<0.01, ** significant difference from control groups, p<0.05.  **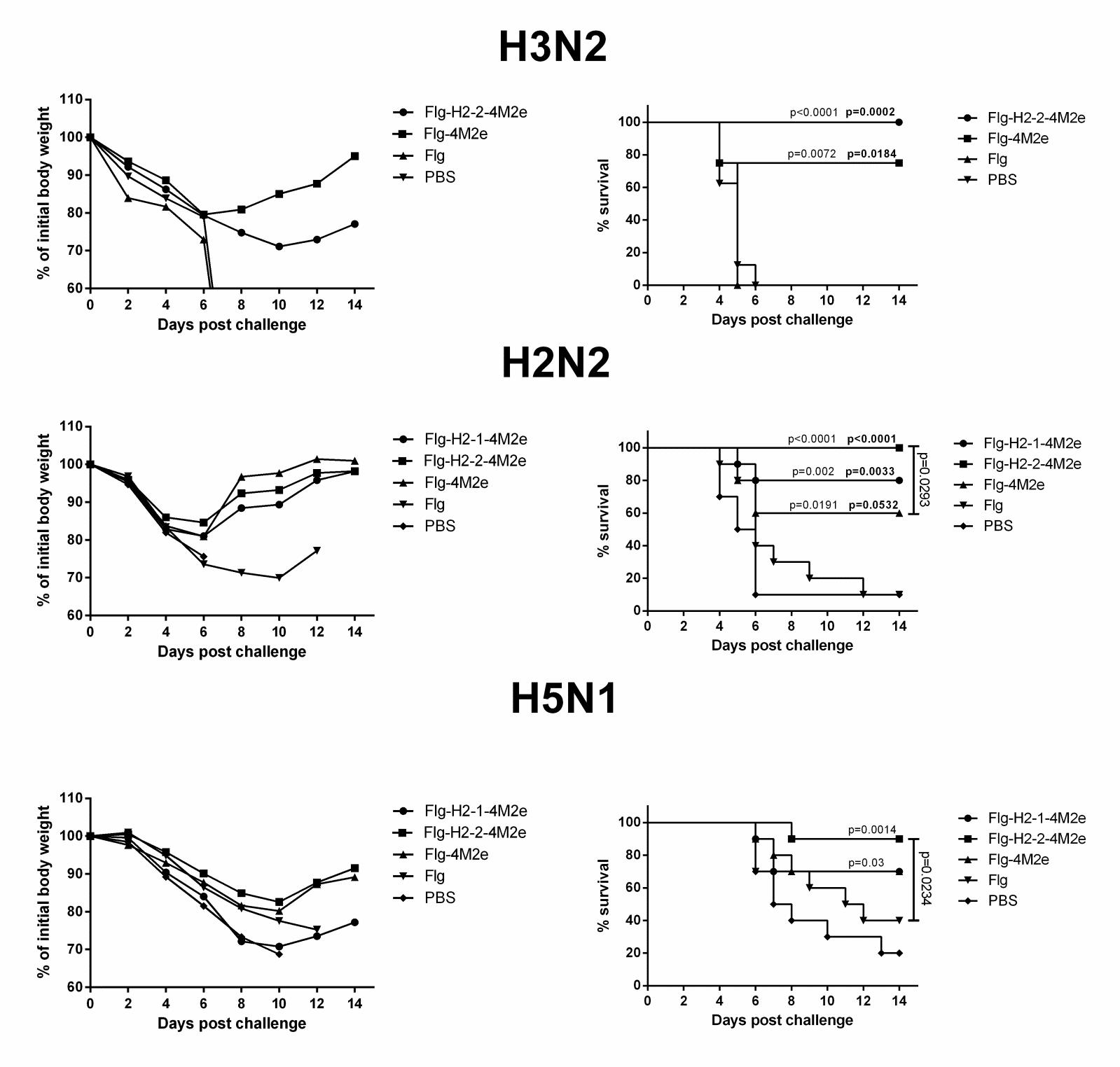**  C  **A B**  Figure 5. M2e specific T-cell response in spleen.  BALB/c mice (n=3/group) were immunized i.n. with 50 μg of Flg-2M2eh2M2ek recombinant protein on days 0, 14, 28. Splenocytes were isolated from 3 mice of each group at day 14 post second boost and assayed for a M2e-stimulated proliferation (A) and M2e specific CD4^+^ T cell response (B). IS ranged in immunized mice from 1,51 to 1,89 (mean=1,69) and in PBS group ranged from 0,79 to 1,35 (mean=1,02). Percent of IL-4+ CD3+CD4+ cells ranged in immunized mice from 0,13 to 0,16 (mean=0,14) and in PBS group ranged from 0,07 to 0,11 (mean=0,09).  Data are presented as the mean±SEM. The index of stimulation (IS) was calculated using the following equation: OD of M2e-treated cells/OD of untreated cells. Statistical significance was determined using Mann-Whitney U-test. The P values between immunized and control group are indicated.  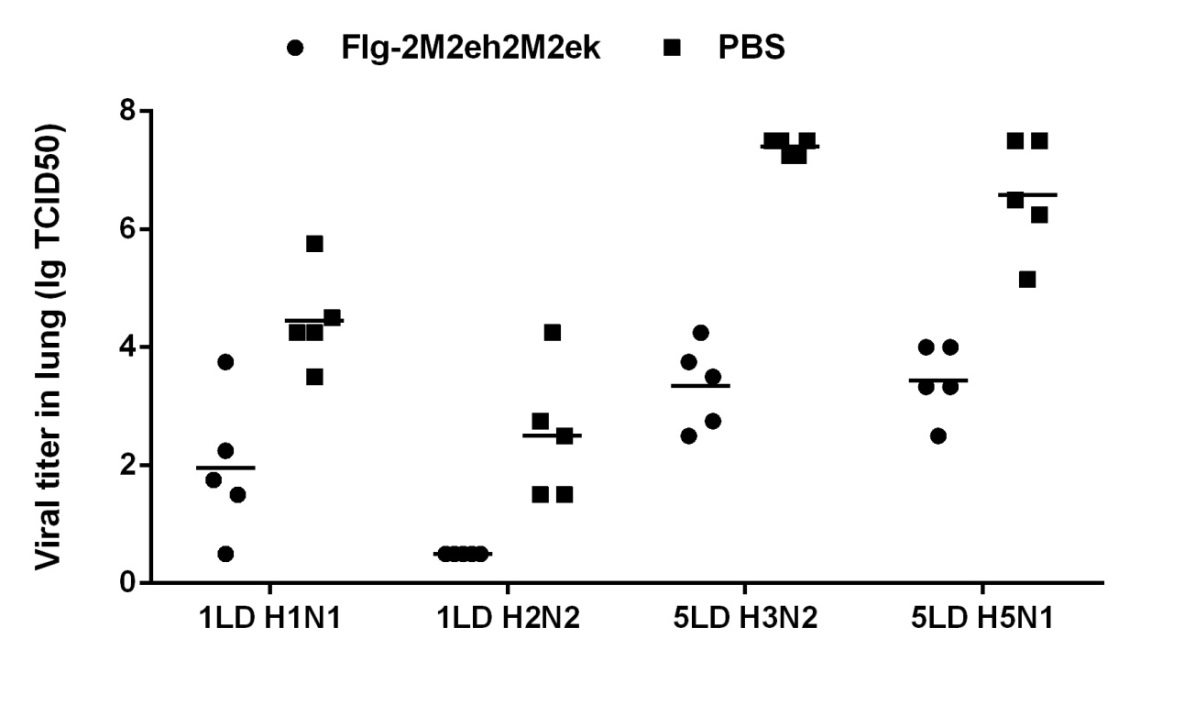  Figure 7. Detection of viral titer in mouse lung.  Mice (n=5/group) immunized with Flg-2M2eh2M2ek fusion peptide were i.n. challenged with 1LD_50_ А/PR/8/34 (H1N1), 1LD_50_ А/Singapore/1/57 (Н2N2), 5LD_50_ А/Aichi/2/68 (H3N2), 5LD_50_ А/Kurgan/5/05 RG (H5N1) and viral titers were detected 6 days post challenge. The data are expressed as lg TCID_50._ Horizontal bars indicate mean among 5 mice per group. The lower limit of detection is 0,5 lg TCID_50._ Statistical significance was determined using Mann-Whitney U-test. The P values between immunized and control group are indicated. Viral titers in lung in immunized mice range from 0.75 to 3.15 (mean=1.95) post challenge with A/H1N1, from 2.64 to 4.06 (mean=3.35) post challenge with A/H3N2, from 0.5 to 0.5 (mean=0.5) post challenge with A/H2N2, from 4.37 to 5.13 (mean=4.75) post challenge with A/H5N1. Viral titers in lung in PBS group range from 4.41 to 4.85 (mean=4.45) post challenge with A/H1N1, from 7.27 to 7.53 (mean=7.4) post challenge with A/H3N2, from 1.5 to 3.5 (mean=2.5) post challenge with A/H2N2, from 5.6 to 7.56 (mean=6.58) post challenge with A/H5N1. |
|  | 17 a | **Fig 7.** **Efficacy of immunization**. Groups of 10 Balb/c mice were immunized with fusion proteins Flg-HA2-2-4M2e, Flg-HA2-1-4M2e or Flg-4M2e (10 μg/0.02 ml). Mice of control groups were administered with Flg-his (10 μg/0.02 ml) or PBS. Two weeks post-second boosting mice were challenged with viruses (**A**) 10LD50 A/Aichi/2/68 (H3N2), (**B**) 10LD50 A/ California/1/66 (H2N2) or **(C)** 5LD50 A/Kurgan/05/05 RG (H5N1). Body weight (left) and survival rate (right) were monitored daily during 14 days. The P values (Montel-Cox test) between immunized and control groups, and between groups immunized with different proteins are indicated (regular font – difference from PBS group; bold font – difference from Flg group).  There were no adverse events after immunization in each experimental group. |
|  | b | There were no modifications to the experimental protocol. |
| DISCUSSION | | |
| Interpretation/ scientific implications | 18 a | In this study, we analyzed three recombinant proteins in terms of their immunogenicities and their abilities to elicit protective responses. We have shown that the introduction of the aa 76-130 polypeptide into the recombinant Flg4M2e protein led to the formation of anti-virus IgG antibodies, in addition to the anti-M2e IgG antibodies induced by the simpler Flg-4M2e protein. Both of the vaccine candidates (Flg-HA2-2-4M2e and Flg-HA2-1-4M2e) were cross-reactive and induced the production of antibodies against viruses from a different phylogenetic group. The observed cross-reactivity is probably due to the presence of monoclonal neutralizing antibodies such as F16 and MED18852, directed against conserved epitopes of HA from both phylogenetic groups. Broadly protective immunity against influenza can be provided by effector memory CD4 and CD8 T cells in lung. Our results show that intranasal immunization of mice with the Flg-HA2-2-4M2e and Flg-4M2e proteins stimulated effector memory M2e-specific CD4+ T-cells in lung (predominantly single-producers of TNF-α) in comparable quantities while immunization with Flg-HA2-1-4M2e stimulated M2e-specific memory CD4+ T-cells in a much lesser quantity. It have been demonstrated that a stronger T-cell immune response and a more robust protective effect were obtained after immunization with recombinant protein containing the HA2-2 fragment. When challenged with a 10LD_50_ load of viruses belonging to both phylogenetic groups, immunized animals demonstrated a strong immune response which kept them alive in 90-100% of cases. |
|  | b | - |
|  | c | - |
| Generalisability/translation | 19 | The study of candidate vaccines in mice is a first step in developing vaccines for human use. |
| Finding | 20 | This work was supported by the Russian Science Foundation (Project No. 15-14-00043). |
|  |  |  |
