# Supplementary material for: Combination of M2e peptide with stalk HA epitopes of influenza A virus enhances protective properties of recombinant vaccine
Source: PLoS One. 2018 Aug 23;13(8):e0201429. doi: 10.1371/journal.pone.0201429 (PMC6107133; doi:10.1371/journal.pone.0201429)
Supplement: S1 Fig — Consensus H1: seasonal human influenza A/H1N1 viruses. Consensus H1pdm: pandemic human influenza A/H1N1pdm viruses. Consensus H2: human and bird influenza viruses of the subtype H2N2. Consensus H5: avian influenza viruses, including those isolated from humans, subtype H5N1. The start of the HA2 subunit is indicated by the arrow; sequences of HA2(76–130) are underlined in red; identical sequences are shown in yellow; substitutions by amino acids similar in properties are shown in green; amino acid substitutions are marked no color; insertions are shown in blue. (DOCX) [file pone.0201429.s002.docx]

1 50

Consensus H3 (1) MKTIIALSYILCLVFAQKLPXGNDNSTATLCLGHHAVPNGTLVKTITNDQ

Consensus H7 (1) -----MNTQILVFALCAIIP----TNADKICLGHHAVSNGTKVNTLTERG

51 100

Consensus H3 (51) IEVTNATELVQSSSTGRICDSPHQILDGENCTLIDALLGDPHCDGFQNKE

Consensus H7 (42) VEVVNATETVERTNIPRICSKGKRTVDLGQCGLLGTITGPPQCDQFLEFS

101 150

Consensus H3 (101) WDLFVERSKAYSNCYPYDVPDYASLRSLVASSGTLEFNNESFNWTGVAQN

Consensus H7 (92) ADLIIERREGSDVCYPGKFVNEEALRQILRESGGIDKESMGFTYSGIRTN

151 200

Consensus H3 (151) GTSSACKRRSVKSFFSRLNWLHX--LKYKYPALNVTMPNNEKFDKLYIWG

Consensus H7 (142) GATSACRR-SGSSFYAEMKWLLSNTDNAAFPQMTKSYKNTRKKPALIIWG

201 250

Consensus H3 (199) VHHPSTDSDQISLYAQASGRVTVSTKRSQQTVIPNIGSRPWVRGVSSRIS

Consensus H7 (191) IHHSGSTTEQTKLYGSGNKLITVGSSNYQQSFVPSPGARPQVNGQSGRID

251 300

Consensus H3 (249) IYWTIVKPGDILLINSTGNLIAPRGYFKIRSGKSSIMRSDAPIGKCNSEC

Consensus H7 (241) FHWLMLNPNDTVTFSFNGAFIAPDRASFLRGKSMGIQSGVQVDANCEGDC

301 350

Consensus H3 (299) ITPNGSIPNDKPFQNVNRITYGACPRYVKQNTLKLATGMRNVPEKQ----

Consensus H7 (291) YHSGGTIISNLPFQNINSRAVGKCPRYVKQESLLLATGMKNVPEIPKGSR

351↓ 400

Consensus H3 (345) -TRGIFGAIAGFIENGWEGMVDGWYGFRHQNSEGTGQAADLKSTQAAIXQ

Consensus H7 (341) VGRGLFGAIAGFIENGWEGLIDGWYGFRHQNAQGEGTAADYKSTQSAIDQ

401 450

Consensus H3 (394) INGKLNRLIEKTNEKFHQIEKEFSEVEGRIQDLEKYVEDTKIDLWSYNAE

Consensus H7 (391) ITGKLNRLIEKTNQQFELIDNEFNEVEKQIGNVINWTRDSMTEVWSYNAE

451 500

Consensus H3 (444) LLVALENQHTIDLTDSEMNKLFERTRKQLRENAEDMGNGCFKIYHKCDNA

Consensus H7 (441) LLVAMENQHTIDLADSEMNKLYERVKRQLRENAEEDGTGCFEIFHKCDDD

501 550

Consensus H3 (494) CIGSIRNGTYDHDVYRDEALNNRFQIKGVELKSGYKDWILWISFAISCFL

Consensus H7 (491) CMASIRNNTYDHSKYREEAMQNRIQIDPVKLSSGYKDVILWFSFGASCFI

551 574

Consensus H3 (544) LCVVLLGFIMWACQKGNIRCNICI

Consensus H7 (541) LLAIAMGLVFICVKNGNMRCTICI
