# Supplementary material for: Combination of M2e peptide with stalk HA epitopes of influenza A virus enhances protective properties of recombinant vaccine
Source: PLoS One. 2018 Aug 23;13(8):e0201429. doi: 10.1371/journal.pone.0201429 (PMC6107133; doi:10.1371/journal.pone.0201429)
Supplement: S2 Fig — Consensus H3: human influenza viruses A/H3N2. Consensus H7: influenza viruses of subtype H7, including those isolated from humans. The start of the HA2 subunit is indicated by the arrow; sequences of HA2 (76–130) are underlined in red; identical sequences are shown in yellow; substitutions by amino acids similar in properties are shown in green; amino acid substitutions are marked no color; insertions are shown in blue. (DOCX) [file pone.0201429.s003.docx]

1 50

Consensus_H1 (1) -----MKAKLLVLLCTFTATYADTICIGYHANNSTDTVDTVLEKNVTVTH

Consensus_H1v (1) -----MXAILVVLLYTFATANADTLCIGYHANNSTDTVDTVLEKNVTVTH

Consensus_H2 (1) XXXXXXXMTITFLILLFTVVKGDQICIGYHANNSTEKVDTILERNVTVTH

Consensus_H5 (1) ----XXMEKIVLLLAIVSLVKSDQICIGYHANNSTEQVDTIMEKNVTVTH

51 100

Consensus_H1 (46) SVNLLEDSHNGKLCXLKGIAPLQLGNCSVAGWILGNPECELLISKESWSY

Consensus_H1v (46) SVNXLEDKHNGKLCKLRGVAPLHLGKCNIAGWILGNPECESLSTASSWSY

Consensus_H2 (51) AKDILEKTHNGKLCRLSGIPPLELGDCSIAGWLLGNPECDRLLSVPEWSY

Consensus_H5 (47) AQDILEKTHNGKLCDLXGVKPLILRDCSVAGWLLGNPMCDEFLNVPEWSY

101 150

Consensus_H1 (96) IVETPNPENGTCYPGYFADYEELREQLSSVSSFERFEIFPKASSWPNHTV

Consensus_H1v (96) IVETSSSDNGTCYPGDFIDYEELREQLSSVSSFERFEIFPKTSSWPNHDS

Consensus_H2 (101) IVEKENPVNGLCYPGSFNDYEELKHLLTSVTHFEKVKILPRDQWTQHTTT

Consensus_H5 (97) IVEKXNPVNDLCYPGDFNDYEELKHLLSRTNHFEKIQIIPXKSSWSNHDA

151 200

Consensus_H1 (146) TKGVTASCSHN-GKSSFYRNLLWLTXKNGLYPNLSKSYXNNKEKEVLVLW

Consensus_H1v (146) NKGVTAACPHA-GAKSFYKNLIWLVKKGNSYPKLSKSYINDKGKEVLVLW

Consensus_H2 (151) GGSRACAVSGN---PSFFRNMVWLTKKGSNYPIAKRSYNNTSGEQMLIIW

Consensus_H5 (147) SSGVSSACPYNXGRSSFFRNVVWLIKKNNAYPTIKRSYNNTNQEDLLVLW

201 250

Consensus_H1 (195) GVHHPPNIGDQRALYHTENAYVSVVSSHYSRRFTPEIAKRPKVRDQEGRI

Consensus_H1v (195) GIHHPSTSADQQSLYQNADAYVFVGXSRYSKKFKPEIAIRPKVRXQEGRM

Consensus_H2 (198) GIHHPNDDAEQRTLYQNVGTYVSVGTSTLNKRSIPEIATRPKVNGQGGRM

Consensus_H5 (197) GIHHPNDAAEQTKLYQNPTTYISVGTSTLNQRLVPXIATRPKVNGQSGRM

251 300

Consensus_H1 (245) NYYWTLLEPGDTIIFEANGNLIAPWYAFALSRGFGSGIITSNAPMXECDA

Consensus_H1v (245) NYYWTLVEPGDKITFEATGNLVVPRYAFAMERNAGSGIIISDTPVHDCNT

Consensus_H2 (248) EFSWTLLETWDVINFESTGNLIAPEYGFKISKRGSSGIMKTEKTLENCET

Consensus_H5 (247) EFFWTILKPNDAINFESNGNFIAPEYAYKIVKKGDSAIMKSELEYGNCNT

301 350

Consensus_H1 (295) ---KCQTPQGAINSSLPFQNVHPVTIGECPKYVRSTKLRMATGLRNIPSI

Consensus_H1v (295) ---TCQTPKGAINTSLPFXNIHPITIGKCPKYVKSTKLRLATGLRNVPSI

Consensus_H2 (298) ---KCQTPLGAINTTLPFHNIHPLTIGECPKYVKSDRLVLATGLRNVPQI

Consensus_H5 (297) XXXKCQTPMGAINSSMPFHNIHPLTIGECPKYVKSNRLVLATGLRNSPQR

351 ↓ 400

Consensus_H1 (342) Q-----SRGLFGAIAGFIEGGWTGMIDGWYGYHHQNEQGSGYAADQKSTQ

Consensus_H1v (342) Q-----SRGLFGAIAGFIEGGWTGMVDGWYGYHHQNEQGSGYAADLKSTQ

Consensus_H2 (345) E-----SRGLFGAIAGFIEGGWQGMVDGWYGYHHSNDQGSGYAADKESTQ

Consensus_H5 (347) ERRRKKXRGLFGAIAGFIEGGWQGMVDGWYGYHHSNEQGSGYAADKESTQ

401 450

Consensus_H1 (387) NAIXGITNKVNSVIEKMNTQFTAVGKEFNXLERRIENLNKKVDDGFLDIW

Consensus_H1v (387) NAIDXITNKVNSVIEKMNTQFTAVGKEFNHLEKRIENLNKKVDDGFLDIW

Consensus_H2 (390) KAIDGITNKVNSVIEKMNTQFEAVGKEFNNLERRLENLNKKMEDGFLDVW

Consensus_H5 (397) KAIDGVTNKVNSIIDKMNTQFEAVGREFNNLERRIENLNKKMEDGFLDVW

451 * * 500

Consensus_H1 (437) TYNAELLVLLENERTLDFHDSNVKNLYEKVKSQLKNNAKEIGNGCFEFYH

Consensus_H1v (437) TYNAELLVLLENERTLDYHDSNVKNLYEKVRSQLKNNAKEIGNGCFEFYH

Consensus_H2 (440) TYNAELLVLMENERTLDFHDSNVKNLYDKVRMQLRDNAKEIGNGCFEFYH

Consensus_H5 (447) TYNAELLVLMENERTLDFHDSNVKNLYDKVRLQLRDNAKELGNGCFEFYH

501 550

Consensus_H1 (487) KCXDECMESVKNGTYDYPKYSEESKLNREXIDGVKLESMGVYQILAIYST

Consensus_H1v (487) KCDNTCMESVKNGTYDYPKYSEEAKLNREEIDGVKLESTRIYQILAIYST

Consensus_H2 (490) KCDDECMNSVRNGTYDYPKYEEESKLNRNEIKGVKLSNMGVYQILAIYAT

Consensus_H5 (497) KCDNECMESVRNGTYDYPQYSEEARLNREEISGVKLESMGTYQILSIYST

551 591

Consensus_H1 (537) VASSLVLL-VSLGAISFWMCSNGSLQCRICIXXXXXXXXXX

Consensus_H1v (537) VASSLVLV-VSLGAISFWMCSNGSLQCRICI----------

Consensus_H2 (540) VAGSLSLA-IMIAGISFWMCSNGSLQCRICI----------

Consensus_H5 (547) VASSLALAXIMVAGLSLWMCSNGSLQCRICIXXXXXXXXX-
