# Supplementary figures and images for: Combination of M2e peptide with stalk HA epitopes of influenza A virus enhances protective properties of recombinant vaccine
Source: PLoS One. 2018 Aug 23;13(8):e0201429. doi: 10.1371/journal.pone.0201429 (PMC6107133; doi:10.1371/journal.pone.0201429)

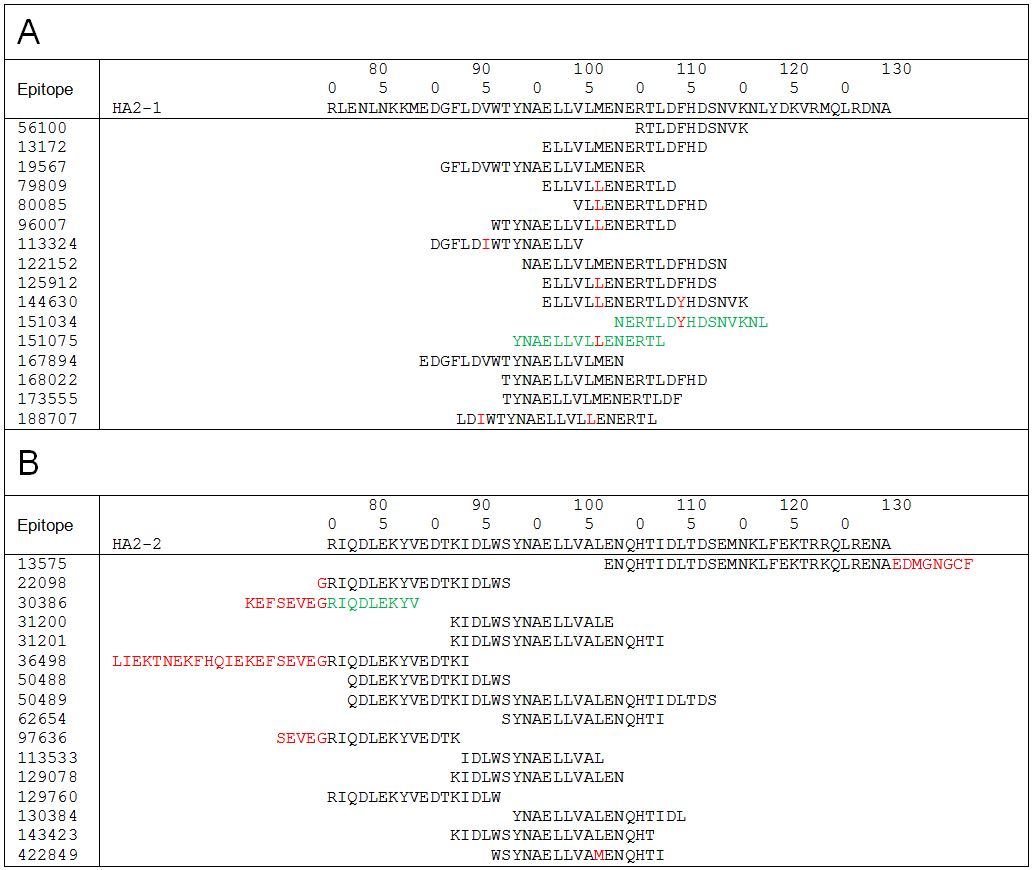

Supplement: S3 Fig — Result of IEDB database search is presented for sequence of influenza viruses from phylogenetic groups I (A) and II (B). Non-homologous amino acids are marked with red. Green font identifies the single B-cell epitope. Black font identifies the CD4+ T-cells epitopes. (TIF) [file pone.0201429.s004.tif]

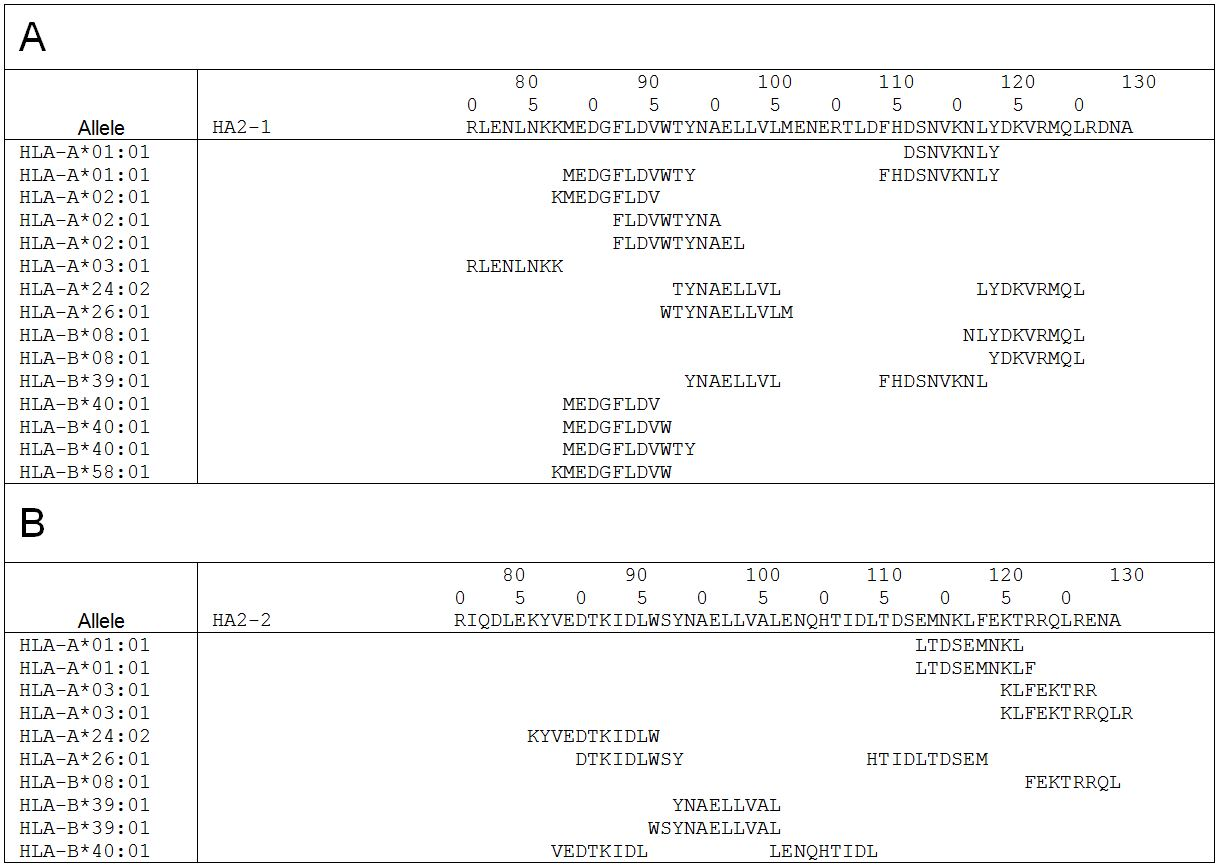

Supplement: S4 Fig — Blue font identifies the CD8+ T-cells epitopes. (TIF) [file pone.0201429.s005.tif]
